# Effect of Point-of-Care Testing for Respiratory Pathogens on Antibiotic Use in Children: A Randomized Clinical Trial

**DOI:** 10.1001/jamanetworkopen.2022.16162

**Published:** 2022-06-09

**Authors:** Suvi Mattila, Niko Paalanne, Minna Honkila, Tytti Pokka, Terhi Tapiainen

**Affiliations:** 1 Department of Pediatrics and Adolescent Medicine, Oulu University Hospital, Oulu, Finland; 2 PEDEGO (Pediatrics, Dermatology, Gynecology, Obstetrics) Research Unit and Medical Research Centre Oulu, University of Oulu, Oulu, Finland; 3 Biocenter Oulu, University of Oulu, Finland

## Abstract

**Importance:**

Limited data are available on the clinical impact of multiplex polymerase chain reaction (PCR) point-of-care testing for respiratory pathogens in acutely ill children.

**Objective:**

To evaluate the effect of multiplex PCR point-of-care testing for respiratory pathogens on antibiotic use in acutely ill children.

**Design, Setting, and Participants:**

This unblinded, randomized clinical trial was conducted from May 6, 2019, through March 12, 2020. The participants were followed up until hospitalization or discharge from the emergency department (ED) for primary outcome. The study was conducted at the pediatric ED of Oulu University Hospital, Finland. Eligible study participants were children aged 0 to 17 years with fever and/or any respiratory signs or symptoms. Children with underlying medical conditions were included. The statistical analyses were performed between August 11, 2020, and September 14, 2021.

**Interventions:**

The participants were randomly assigned in a 2:1 ratio either to undergo multiplex PCR point-of-care testing (18 respiratory viruses and 3 bacteria with results ready within 70 minutes) upon arrival at the ED or to receive routine care.

**Main Outcomes and Measures:**

The primary outcome was the proportion of children receiving antibiotic therapy. The secondary outcomes were the numbers of diagnostic tests and radiographic imaging procedures performed and costs.

**Results:**

A total of 1417 children were assessed for eligibility. After exclusions, 1243 children (692 boys [56%]) were randomly allocated to either the intervention (829 children) or control (414 children) group. The mean (SD) age of the participants was 3.0 (3.6) years in the intervention group (median [IQR], 1.7 [0.4-4.1] years) and 3.0 (3.5) years (median [IQR], 1.9 [0.4-4.1] years) in the control group. Multiplex PCR point-of-care testing for respiratory pathogens did not reduce the overall prescribing of antibiotics in the emergency department (226 children [27.3%] in the intervention group vs 118 children [28.5%] in the control group; risk ratio, 0.96; 95% CI, 0.79-1.16). Targeted antibiotic therapy was started in 12 children (1.4%) tested with point-of-care multiplex PCR and 2 children (0.5%) in the control group (risk ratio, 3.0; 95% CI, 0.76-11.9). The numbers of diagnostic tests did not differ between the groups, nor did the costs.

**Conclusions and Relevance:**

In this randomized clinical trial, point-of-care testing for respiratory pathogens did not reduce the use of antibiotics at the pediatric ED. Testing for respiratory pathogens appears to have a limited impact on clinical decision-making for acutely ill children.

**Trial Registration:**

ClinicalTrials.gov Identifier: NCT03932942

## Key Points

**Question:**

Does point-of-care testing for respiratory pathogens reduce antibiotic use in acutely ill children?

**Findings:**

In this randomized clinical trial of 1243 children, multiplex polymerase chain reaction point-of-care testing for respiratory pathogens did not reduce the overall prescribing of antibiotics in the emergency department (27.3% in the intervention group vs 28.5% in the control group).

**Meaning:**

These findings suggest that testing for respiratory pathogens does not reduce antibiotic use in acutely ill children.

## Teaser

This randomized clinical trial evaluates the effect of multiplex polymerase chain reaction point-of-care testing for respiratory pathogens on the antibiotic use in acutely ill children.

## Introduction

Acute respiratory tract infections are the main cause of morbidity and emergency department (ED) visits for children. Given that diagnostic uncertainties^1,2,3^ lead to antibiotic treatment for up to 70% of respiratory infections in children^4,5,6^ and that antibiotic consumption is associated with increasing antibacterial resistance,^7,8^ new approaches are needed to guide the prescribing of antibiotics for acutely ill children.

Rapid testing for influenza viruses has been found to reduce the prescribing of antibiotics in EDs,^9,10,11^ the number of ancillary tests and radiological examinations,^11,12^ the length of stay in the ED,^9^ and the associated costs for acutely ill children.^9^ Polymerase chain reaction (PCR) platforms that can simultaneously detect multiple respiratory pathogens within 1 hour have recently been developed and have also been found to be suitable for point-of-care testing in an ED.^13^ Limited data are available, however, on the benefits and clinical impact of multiplex PCR point-of-care testing for respiratory pathogens in acutely ill children. One previous study^14^ involving approximately 200 children with febrile acute respiratory tract infections showed that rapid testing for 7 respiratory viruses reduced the prescription of antibiotics for the same illness within 1 week of discharge from the ED. Having hypothesized that rapid multiplex PCR point-of-care testing for respiratory viral and bacterial pathogens would reduce the prescribing of antibiotics for acutely ill children, we conducted a randomized clinical trial (RCT) to evaluate the clinical impact of such testing in a pediatric ED.

## Methods

### Study Design and Oversight

We assessed the clinical impact of point-of-care testing for multiple respiratory viral and bacterial pathogens in pediatric patients upon arrival at a pediatric ED, with results available within 70 minutes, and compared the outcomes with those obtained with routine clinical care including testing for influenza. Children aged 0 to 17 years with fever (>38.0 °C) and/or any respiratory signs or symptoms (tachypnea, shortness of breath, apnea, wheezing, cough, rhinitis, croup, sneezing, earache, sore throat, or some other suspicion of respiratory infection) were eligible for participation. Exclusion criteria were the need for cardiopulmonary resuscitation or immediate transfer to the intensive care unit. Otherwise, patients with comorbidities were included. All children were from Finnish-speaking families.

This was an investigator-driven academic clinical trial conducted at the Pediatric Emergency Department of Oulu University Hospital, Finland, from May 6, 2019, to March 12, 2020. A point-of-care diagnostic device was acquired by NordLab, Finland, the clinical microbiology laboratory at Oulu University Hospital, under national procurement legislation. Before the initiation of the trial, the pediatric nurses in the ED were trained to use this device, and physicians were advised on interpretation of the results. An educational guideline for the interpretation of pathogen findings was available for physicians (Supplement 1).

The protocol was approved by the Ethical Committee of Oulu University Hospital, Finland. All the data were available to the authors, who vouched for the accuracy and completeness of the information and for staff adherence to the protocol of the trial (Supplement 1). This trial followed the Consolidated Standards of Reporting Trials (CONSORT) reporting guideline for RCTs with extension for pragmatic trials.

### Trial Procedures

The participants were randomly assigned in a 2:1 ratio to undergo either multiplex PCR point-of-care testing upon arrival at the pediatric ED or receive routine care. Those in the intervention group were tested for 18 respiratory viruses or viral subtypes and 3 bacteria, with results ready within 70 minutes, whereas those in the control group were tested with multiplex PCR for respiratory pathogens only according to the clinical judgment of the ED physician, the results being available the next day. Both groups were allowed to undergo other laboratory tests, including a laboratory-based PCR assay to detect influenza A/B and respiratory syncytial virus (RSV), with results ready in 3 hours, and point-of-care C-reactive protein assays, with results ready in 15 minutes.

Pediatric nurses in the ED screened children for eligibility upon arrival. After obtaining written informed consent from each child’s legal guardian and from children older than 6 years, the ED nurse opened the next opaque randomization envelope to assign the participant to either the intervention or control group. Before the study commenced, a biostatistician who was not involved in the data collection had created a randomization list using a computer-generated random sequence of numbers, randomization being performed in permuted blocks of sizes varying randomly between 3, 6, and 9.

### Sample Collection and Pathogen Detection

The pediatric nurses collected nasopharyngeal specimens from each child by passing a flocked swab (FLOQSwabs; Copan Diagnostics, Inc) through the nostril to the nasopharynx and rotating it to gather epithelial cells. Pathogen detection in the intervention group was performed using the QIAstat-Dx (DiagCORE) Respiratory Panel V2 (Qiagen), a multiplex diagnostic test with results available in 70 minutes. At the time of the study, the panel detected the following pathogens: adenovirus, bocavirus, coronavirus 229E, coronavirus HKU1, coronavirus NL63, coronavirus OC43, human metapneumovirus A and B, influenza A, influenza A subtype H1N1/2009, influenza A subtype H1, influenza A subtype H3, influenza B, parainfluenza viruses 1 to 4, RSV A and B, rhinovirus-enterovirus, *Bordetella pertussis*, *Legionella pneumophila*, and *Mycoplasma pneumoniae*. The diagnostic accuracy of the QIAstat-Dx Respiratory Panel V2 has been validated elsewhere.^15^ The swab for pathogen detection was applied to the cartridge, and the results were transferred to the electronic medical record system, which notified the physicians. The protocol allowed for repeated testing in the case of technical errors.

The children in the control group were allowed to be tested with multiplex PCR in the laboratory according to clinical judgment, but the results were available the next day. Thus, the results of multiplex PCR in the control group were not available in the ED, when the clinician decided to start antibiotic treatment.

### Outcomes

The primary outcome was the proportion of children with any antibiotic therapy started or ongoing in the ED or any antibiotic therapy prescribed in the ED. In the exploratory analysis, after the trial was commenced, the proportion of children with an antibiotic prescription was collected and reported separately for untargeted and pathogen-targeted antibiotic therapy. This was done because the effects of the intervention might be divergent for targeted and untargeted antibiotic therapy, as point-of-care multiplex PCR testing used in the intervention group recognized several bacterial pathogens with targeted antibiotic therapy available. The pathogen-targeted antibiotic therapy was defined as antibiotic therapy against *M pneumoniae*,* L pneumophila*, or* B pertussis* but not against influenza.

The main secondary outcomes were the numbers of diagnostic tests and radiographic imaging procedures performed and costs associated with visits to the ED. Visit associated costs were provided by the KULAS (Kuntalaskutustieto) database, which collects expenses on health care visits and charges these expenses from the municipality where patient lives. Other secondary outcomes were the proportions of children with a macrolide antibiotic prescription in the ED and the mean time for starting targeted therapy for influenza or mycoplasma. The length of stay in the ED was reported. The proportion of children with an antibiotic prescription, hospital admissions, visits to the ED, and outpatient telephone contacts were reported within 1 week of entry. The proportion of children admitted to intensive care and deaths were reported within 1 month of entry.

### Data Collection

The physicians involved in the study reviewed all relevant medical records, including records stored on a nationwide centralized electronic database covering all prescription data and medical record data in Finland.^16^ Data on laboratory tests performed, length of stay, and associated costs were obtained from a hospital database.

### Sample Size

Before the study in 2015, in a sample of 1195 children treated at the pediatric ED, antibiotics were administered to 31% (95% CI, 28%-34%) of the patients. Since we assumed that approximately 33% of the children in the control group would receive antibiotics and considered a relative reduction of 25% to be clinically significant, we estimated that, with an α error of 5% and a statistical power of 80% and using 2:1 randomization, we needed 785 case patients and 392 controls, for a total of 1177 children. We planned to recruit participants for 1 epidemiological year, from May 2019 to May 2020. In practice, however, the trial had to be discontinued 7 weeks earlier than planned because of the COVID-19 pandemic. On the other hand, by that time, we had recruited and randomized 1350 children.

### Statistical Analysis

All the statistical analyses were performed according to the intention-to-treat principle. The primary and secondary outcomes were analyzed by calculating the 95% CIs of the differences using a standard normal deviate test for proportions^17^ as well as a *t* test for continuous variables. Proportions, proportion differences, and risk ratios (RRs) with 95% CIs were calculated for categorial outcomes. Because the widths of the CIs were not adjusted for multiplicity, the inferences drawn may not be reproducible. Missing data were rare. All the analyses were performed using IBM SPSS Statistics software for Windows version 27.0 (IBM Corp) and StatsDirect statistical software version 3 (StatsDirect Ltd). The statistical analyses were performed between August 11, 2020, and September 14, 2021.

## Results

### Characteristics of the Patients

**Figure 1.**
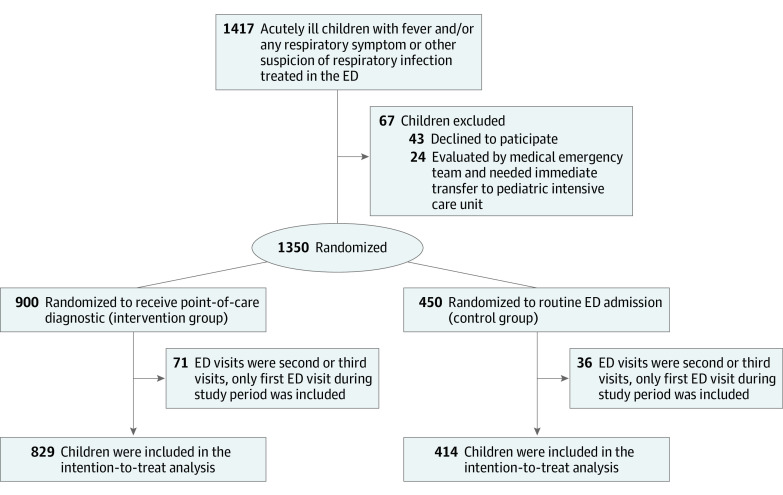
Study Design Flow chart shows recruitment, randomization, and study population. ED indicates emergency department.

A total of 1417 children were assessed for eligibility, including 43 who declined to participate and 24 who needed immediate emergency care, including hemodynamically unstable patients evaluated by medical emergency team (Figure 1). Randomization was thus performed at 1350 visits. A further 107 cases that had been randomized were nevertheless later found to have been revisits by previously recruited participants and were therefore excluded. Eventually, 1243 participants (692 boys [56%]), 829 in the intervention group and 414 in the control group, formed the intention-to-treat population. The mean (SD) age of the participants was 3.0 (3.6) years (median [IQR], 1.7 [0.4-4.1] years) in the intervention group and 3.0 (3.5) years (median [IQR], 1.9 [0.4-4.1] years) in the control group (Table 1).

**Table 1.** Baseline Demographic and Clinical Characteristics of the Participants

Characteristic	Patients, No. (%)	
	Intervention group, point-of-care diagnostics on arrival at the ED (n = 829)	Control group, routine ED visit (n = 414)
Age, y		
Mean (SD)	3.0 (3.6)	3.0 (3.5)
Median (IQR)	1.7 (0.4-4.1)	1.9 (0.4-4.1)
Age group, y		
<2	467 (56.3)	222 (53.6)
≥2 to <6	232 (28.0)	128 (30.9)
≥6	130 (15.7)	64 (15.5)
Sex		
Female	366 (44.1)	185 (44.7)
Male	463 (55.9)	229 (55.3)
Inclusion criteria		
Only fever	155 (18.7)	75 (18.1)
Only respiratory tract symptoms	318 (38.4)	154 (37.2)
Fever and respiratory tract symptoms	346 (41.7)	181 (43.7)
Other suspicion of a respiratory tract infection	10 (1.2)	4 (1.0)
Underlying medical condition		
None	621 (74.9)	324 (78.3)
Preterm infant (<37 wk gestation)	37 (4.5)	16 (3.9)
Congenital malformation	27 (3.3)	11 (2.7)
Asthma	26 (3.1)	16 (3.9)
Intellectual disability	17 (2.1)	9 (2.2)
Severe heart disease^a^	11 (1.3)	7 (1.7)
Epilepsy	10 (1.2)	6 (1.4)
Down syndrome	8 (1.0)	4 (1.0)
Chronic respiratory disease	5 (0.6)	2 (0.5)
Diabetes	3 (0.4)	3 (0.7)
Organ transplant	3 (0.4)	2 (0.5)
Malignant neoplasm	2 (0.2)	3 (0.7)
Primary immunodeficiency^b^	2 (0.2)	2 (0.5)
Rheumatoid arthritis	1 (0.1)	1 (0.2)
Any other underlying medical condition	37 (4.4)	21 (5.1)
Diagnostic tests performed		
Multiplex PCR point-of-care test (intervention)	827 (99.8)	6 (1.4)
Multiplex PCR in the laboratory (results available the next day)	0	258 (62.3)
Reverse transcriptase–PCR for influenza and respiratory syncytial virus (results available in 2-3 h)	26 (3.1)	165 (39.9)
C-reactive protein at the point-of-care	572 (69.0)	293 (70.8)
C-reactive protein in the laboratory	135 (16.3)	75 (18.1)
C-reactive protein level, mean (SD), mg/dL	4.2 (6.3)	4.8 (6.6)

Abbreviations: ED, emergency department; PCR, polymerase chain reaction.

SI conversion factor: To convert C-reactive protein to milligrams per liter, multiply by 10.

^a^ Refers to congenital heart defect, heart transplant, or cardiomyopathy.

^b^ In the intervention group, 1 patient had ADA2 deficiency and 1 had hyposplenia; in the control group, 1 patient had hyper-IgE syndrome and 1 had combined immunodeficiency.

In total, 74.9% of the participants in the intervention group (621 children) and 78.3% in the control group (324 children) had no underlying medical conditions. The most common acute diagnoses were viral wheezing or bronchiolitis.

In the control group, 258 participants (62.3%) were tested for respiratory pathogens with multiplex PCR performed in the laboratory, with the results being available the next day. Reverse transcriptase–PCR for influenza and RSV, with results available in 2 to 3 hours, was performed for 26 children (3.1%) in the intervention group and 165 children (39.9%) in the control group. The failure rate after the first attempt was (8%).

**Figure 2.**
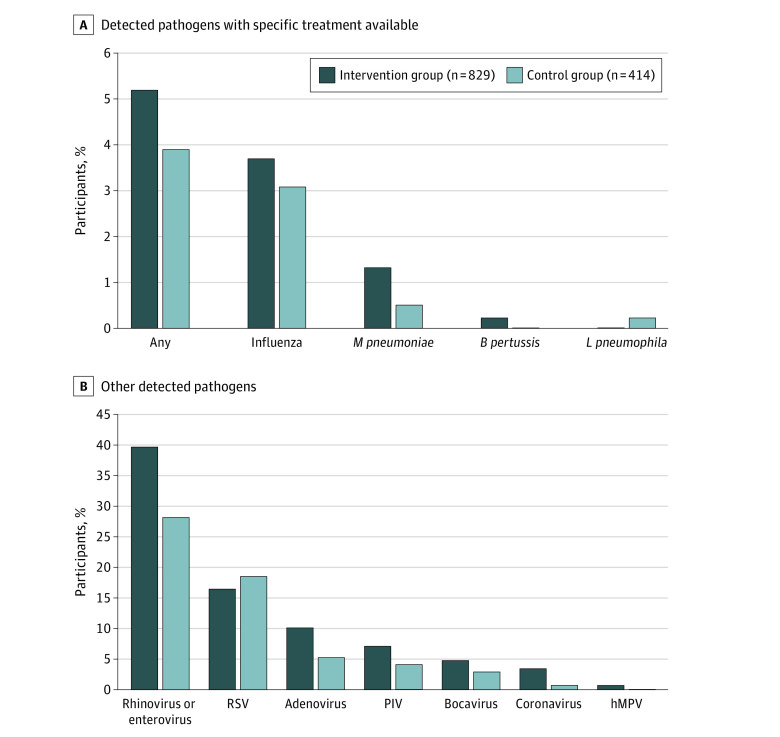
Respiratory Pathogens Detected From the Study Participants In the intervention group, all pathogen results were available within 70 minutes. In the control group, respiratory syncytial virus (RSV) and influenza test results, performed according to clinical judgment, were available within 3 hours. In the control group, multiplex polymerase chain reaction was performed according to clinical judgment with results ready on the next office day. Panel A shows the pathogens with specific treatment available. Panel B shows other detected pathogens. *B pertussis *indicates *Bordetella pertussis;* hMPV, human metapneumovirus; *L pneumophila, Legionella pneumophila; M pneumoniae, Mycoplasma pneumoniae*; PIV, parainfluenza virus.

The indications for prescribing antibiotics were mostly concordant with current national guidelines (Table 2).^18,19^ The most common discharge diagnoses associated with improper antibiotic use (ie, use not recommended in the national guidelines)^19^ were viral wheezing, croup, and viral tonsillitis. The most commonly detected pathogens were rhinovirus-enterovirus, followed by RSV and adenovirus (Figure 2). The detection of rhinovirus-enterovirus peaked in spring and autumn (eFigure in Supplement 2). Thirty-one participants in the intervention group (3.7%) and 13 in the control group (3.1%) were influenza positive.

**Table 2.** Use of Antibiotics Within 7 Days of an ED Visit, According to the Discharge Diagnoses^a^

Diagnoses	Patients, No./total No. (%)	
	Intervention group (n = 829)	Control group (n = 414)
Diagnoses with guideline-concordant antibiotic use^b^		
Any diagnosis recommending antibiotic use	181/190 (95.3)	93/99 (93.9)
AOM	89/93 (95.7)	43/45 (95.6)
Pneumonia	53/56 (94.6)	26/29 (89.7)
Viral wheezing with AOM	30/31 (96.8)	11/12 (91.7)
Urinary tract infection	28/28 (100)	9/9 (100)
* Mycoplasma pneumoniae*^c^	6/6 (100)	0
Sepsis	3/3 (100)	5/5 (100)
Central nervous system infection	2/2 (100)	2/2 (100)
Tonsillitis caused by group A streptococci	2/2 (100)	1/1 (100)
Pertussis	2/2 (100)	0
Lyme disease	1/1 (100)	2/2 (100)
Myocarditis	1/1 (100)	0
Postoperative infection	1/1 (100)	0
Other infection^d^	12/14 (85.7)	12/13 (92.3)
Diagnoses with antibiotic use not recommended in guidelines^b^		
Diagnoses not requiring antibiotic use	102/639 (16.0)	50/315 (15.9)
Unspecified acute respiratory infection	36/199 (18.1)	16/90 (17.8)
Viral wheezing or bronchiolitis without AOM	26/184 (14.1)	6/76 (7.9)
Other viral infection	22/87 (25.3)	6/35 (17.1)
Streptococcal tonsillitis other than group A^e^	18/39 (46.2)	11/24 (45.8)
Fever of unknown origin	11/35 (31.4)	7/27 (25.9)
Croup	5/31 (16.1)	9/28 (32.1)
Febrile convulsions	5/28 (17.9)	7/21 (33.3)
Gastroenteritis	2/17 (11.8)	0
Nonspecific symptoms peculiar to infancy	1/17 (5.9)	0
Kawasaki disease	0	1/1 (100)
Tick-borne encephalitis	0	1/1 (100)
Other diagnoses^f^	10/50 (20.0)	5/28 (17.9)

Abbreviations: AOM, acute otitis media; ED, emergency department.

^a^ Refers to diagnosis at discharge from the ED or hospital ward.

^b^ Refers to proportion of participants receiving an antibiotic prescription within each diagnostic class. One participant could have up to 3 discharge diagnoses.

^c^ Six participants in the intervention group had pneumonia caused by *Mycoplasma pneumoniae*.

^d^ Other infections included balanitis, abscess, lymphadenitis, parotitis, skin infection, cellulitis, conjunctivitis, sinusitis, stomatitis, tularemia, and facial paresis with an infectious cause.

^e^ Refers to adenovirus infection or other undefined tonsillitis. Two participants in the intervention group and 1 in the control group had tonsillitis caused by group A β-hemolytic streptococci. The diagnosis was confirmed by a rapid antigen detection test.

^f^ Refers to any other noninfectious discharge diagnoses.

### Primary Outcome

In the intention-to-treat population, multiplex PCR point-of-care testing for respiratory pathogens did not reduce the overall prescribing of antibiotics in the ED (226 children [27.3%] in the intervention group vs 118 children [28.5%] in the control group; RR, 0.96; 95% CI, 0.79 to 1.16). Additional findings are shown in Table 3.

**Table 3.** Trial Outcomes for the Intention-to-Treat Population

Outcomes	Patients, No./total No. (%)		Difference, mean (95% CI)	Risk ratio (95% CI)
	Intervention group (n = 829)	Control group (n = 414)		
Primary outcome, antibiotic prescription in the ED^a^	226/829 (27.3)	118/414 (28.5)	–1.2 (–6.6 to 4.0)	0.96 (0.79 to 1.16)
Untargeted antibiotic prescription in the ED	214/829 (25.8)	116/414 (28.0)	–2.2 (–7.6 to 2.9)	0.92 (0.76 to 1.12)
Pathogen-targeted antibiotic prescription in the ED	12/829 (1.4)	2/414 (0.5)^b^	0.96 (–2.1 to 0.4)	3.0 (0.76 to 11.9)
Secondary outcomes				
Antibiotic prescription within 1 week	283/829 (34.1)	143/414 (34.5)	–0.4 (–6.1 to 5.1)	0.99 (0.84 to 1.17)
Macrolide antibiotic in the ED	14/829 (1.7)	6/414 (1.4)	0.2 (–1.6 to 1.6)	1.17 (0.47 to 2.92)
Macrolide in infants aged <3 mo	1/175 (0.6)	3/85 (3.5)	–3.0 (–9.4 to 0.3)	0.16 (0.02 to 1.12)
Hospital admissions at an ED visit	346/829 (41.7)	177/414 (42.8)	–1.0 (–6.9 to 4.8)	0.98 (0.85 to 1.12)
Hospital admissions within 1 wk of an ED visit^c^	361/829 (43.5)	190/414 (45.9)	–2.3 (–8.2 to 3.5)	0.95 (0.83 to 1.08)
Readmission to the ED within 1 wk^c^	120/829 (14.5)	72/414 (17.4)	–2.9 (–7.5 to 1.3)	0.83 (0.64 to 1.09)
Outpatient telephone contact within 1 week	106/829 (12.8)	69/414 (16.7)	–3.9 (–8.3 to 0.2)	0.77 (0.58 to 1.02)
Chest radiograph in the ED	124/829 (15.0)	65/414 (15.7)	–0.7 (–5.2 to 3.4)	0.95 (0.73 to 1.26)
Any radiographic imaging within 1 wk	242/829 (29.2)	122/414 (29.5)	–0.3 (–5.7 to 5.0)	0.99 (0.83 to 1.19)
Admission to pediatric intensive care within 30 d	21/829 (2.5)	9/414 (2.2)	0.4 (–1.7 to 2.0)	1.17 (0.55 to 2.48)
Deaths within 30 d	0	0	NA	NA
Total costs, €^d^	994.7	831.5	163.2 (–120.0 to 446.3)	NA
Laboratory tests in the ED, mean (SD), No. per participant	5.1 (4.5)	5.1 (4.2)	–0.04 (–0.6 to 0.5)	NA
Length of stay in the ED, mean (SD), min	182.6 (80.6)	169.4 (80.6)	13.2 (3.7 to 22.7)	NA
Time to oseltamivir if influenza, mean (SD), min	886 (1611)	977 (1349)	–92 (–1148 to 1332)	NA
Time to macrolide if *Mycoplasma pneumoniae*, mean (SD), min	510 (348)	2515 (2808)	–2005 (–26 900 to 22 900)	NA
Time to macrolide if *Bordetella pertussis*, mean (SD), min	223 (109)	NA	NA	NA
Explanatory post hoc analyses, duration of therapy, mean (SD), d^e^	2.5 (3.9)	2.6 (4.0)	0.09 (– 0.38 to 0.55)	NA

Abbreviations: ED, emergency department; NA, not applicable.

^a^ Ongoing antibiotic treatment on arrival at the ED was interpreted as a new antibiotic prescription if there was no mention in the medical records of any discontinuation of antibiotic treatment. Only antibacterial agents were defined as antibiotics.

^b^ Two children received pathogen-targeted therapy in the control group: the first child received multiplex polymerase chain reaction point-of-care testing because of a randomization error (ie, the envelope included instructions for both groups), whereas the respiratory specimen of the second child was immediately delivered to the laboratory by mistake.

^c^ Data on hospital admissions and readmissions to the ED were missing for 1 participant in the control group.

^d^ Data on costs associated with the visit were available for 645 participants in the intervention group and 345 participants in the control group. The costs of a diagnostic cartridge (90.47 €) were added to the expenses in the intervention group. As of May 4, 2022, 1 € = $1.06 US.

^e^ Days of therapy up to 7 days after ED visit represents the count of the number of individual antibiotic agents given to a patient on each calendar day regardless of the number of doses.

### Secondary Outcomes

The numbers of diagnostic tests or instances of radiographic imaging did not differ between the groups, nor did the costs of ED visits (Table 3). Multiplex PCR point-of-care testing for respiratory pathogens did not have a significant effect on the prescribing of antibiotics within 1 week of entry (Table 3). The proportion of children who were prescribed macrolide antibiotics in the ED did not differ between the groups when all the children or only the infants younger than 3 months were considered. The mean time to the commencement of targeted treatment for influenza or mycoplasma did not differ between the intervention and control groups.

The length of stay in the ED was longer in the intervention group than in the control group (182.6 minutes vs 169.4 minutes; mean difference, 13.2 minutes; 95% CI, 3.7-22.7 minutes). The proportion of children with hospital admissions, revisits to the ED, outpatient telephone contacts, or admissions to pediatric intensive care did not differ between groups. There were no deaths in the study population within 1 month of entry.

### Exploratory Analyses

The proportion of children who received untargeted antibiotic therapy was 25.8% (214 children) in the intervention group and 28.0% (116 children) in the control group (RR, 0.92; 95% CI, 0.76 to 1.12). The proportion of children who received pathogen-targeted antibiotic therapy was 1.4% (12 children) in the intervention group and 0.5% (2 children) in the control group (RR, 3.0; 95% CI, 0.76 to 11.9). The mean (SD) number of days of antibiotic therapy was 2.5 (3.9) in the intervention group and 2.6 (4.0) in the control group (mean difference, 0.09; 95% CI, –0.38 to 0.55) (Table 3). When patients tested for influenza and RSV in the control group were excluded, no significant difference was found for the primary outcome (eTable 1 in Supplement 2). In total, 83 of 829 participants (10.0%) in the intervention group and 60 of 414 participants (14.5%) in the control group were discharged from the ED within 90 minutes after study entry and had no PCR results available at the time (eTable 1 in Supplement 2). When these participants were excluded, no significant difference was found in primary outcome (eTable 1 in Supplement 2).

## Discussion

In this RCT of 1243 children with fever or respiratory symptoms, multiplex PCR point-of-care testing for respiratory pathogens did not reduce the overall prescribing of antibiotics in the emergency department. In the exploratory analysis, targeted antibiotic therapy was started for 1.4% of children tested with point-of-care multiplex PCR for respiratory pathogens in the ED. Point-of-care multiplex PCR testing in the ED did not, however, decrease the untargeted antibiotic consumption compared with routine care.

The causes of respiratory tract infections in children are difficult to determine on the basis of clinical signs and symptoms.^20,21,22^ There have been 2 earlier RCTs investigating the impact of testing for multiple respiratory pathogens in acutely ill children (eTable 2 and eTable 3 in Supplement 2).^14,23,24,25,26^ Rapid testing of 204 children for 7 respiratory viruses using a direct immunofluorescence assay significantly reduced the prescription of antibiotics after discharge from the ED,^14^ although, in a recently published RCT involving 908 children with influenza-like illnesses, the use of rapid respiratory pathogen testing in the pediatric ED did not reduce the overall prescribing of antibiotics.^27^ Moreover, the accompanying intention-to-treat analyses showed that children subjected to rapid multipathogen testing were even more likely to receive antibiotics.^27^

The negative results reported in the present study should be interpreted with caution. Rapid laboratory-based diagnostic testing for influenza and RSV was performed in the control group. Before conducting the study, we considered the option to stop other respiratory viral testing in the ED except point-of-care multiplex PCR testing in the intervention group. We deemed this approach to be unethical because influenza testing has previously been shown to be beneficial in children.^9,10,11,12^ The additional benefits of point-of-care multiplex PCR testing, however, appeared to be limited according to our results.

We observed an increased length of stay in the ED among children tested for respiratory pathogens with point-of-care multiplex PCR. First, it is possible that physicians decided to wait for the PCR results for children in the intervention group. Second, the failure rate reported after the first attempt was higher than expected (8%). In a recent evaluation of the Qiagen respiratory panel,^15^ invalid results or none at all were obtained from 4% of the specimens, whereas the previously published failure rates among other similar PCR testing devices had ranged from 0.7% to 4.8%.^28,29^ The overall costs or the number of other diagnostics tests did not increase in the children tested with a point-of-care device in the ED in the present study.

The strengths of the present study include the fact that broad respiratory multiplex PCR panels, including those detecting SARS-CoV-2, have now become available for clinical use, making the topic particularly relevant.^30^ This study is one of the first RCTs to outline the role of diagnostic stewardship in decentralizing laboratory diagnostics and implementing new advanced molecular point-of-care assays in clinical practice. We achieved a sample size sufficient to assess the predefined outcomes and were able to report the effects of multiplex PCR testing on both the targeted and untargeted prescribing of antibiotics.

### Limitations

The main limitation of this study is that the proportion of children who were actively tested for RSV and influenza in the ED was high in the control group, suggesting that the effect of the intervention may differ in centers with less active baseline diagnostics for respiratory pathogens. Our study was conducted in a high-income country with low antibiotic consumption. In Europe, antibiotic prescription rates for febrile children range from 22% to 42%.^31^ Thus, it is unknown whether testing for respiratory pathogens reduces antibiotic use in settings with different antibiotic prescription policies. Most participants were tested for C-reactive protein, which likely has guided clinical care. In addition, interpreting the findings from multiplex PCR is complex. The detection of respiratory virus does not exclude the presence of bacterial infection because viral-bacterial coinfections are common among children.^1^The detection of a respiratory virus does not always explain the acute symptoms since the finding may be coindicental.^32^ We enrolled acutely ill children with an assumed respiratory infection on their arrival at the ED. In selected cohorts, the impact of multiplex PCR testing for respiratory pathogens may be different. The number of children with influenza, *M pneumoniae*, or *B pertussis* was low in the study population. Before the study, the proportion of children who received antibiotics in the ED was a little higher than during the study. The intervention in the ED may have influenced the behavior of physicians regarding the treatment of the control group in response to their awareness of the trial.

## Conclusions

In this RCT of acutely ill children with fever or respiratory symptoms, point-of-care testing for respiratory pathogens did not influence the prescribing of antibiotics compared with routine care. Systematic testing for respiratory pathogens in the ED appears to have a limited impact on clinical decision-making regarding antimicrobial therapy for acutely ill children.

## Notes

Supplement 1.
Trial Protocol

Supplement 2. **eTable 1.** Post Hoc Explanatory Analyses **eTable 2.** Studies on the Clinical Impact of Point-of-Care Diagnostics of Respiratory Pathogens in Acutely Ill Children With Respiratory Symptoms: Study Design, Sample Sizes, Methods and Tested Pathogens of the Previous Studies **eTable 3.** Studies on the Clinical Impact of Point-of-Care Diagnostics of Respiratory Pathogens in Acutely Ill Children With Respiratory Symptoms: Outcomes of the Previous Studies
eReferences
**eFigure.** Seasonality of Respiratory Pathogens During the Study

Supplement 3.
Data Sharing Statement
