# Supplementary material for: Effect of Point-of-Care Testing for Respiratory Pathogens on Antibiotic Use in Children: A Randomized Clinical Trial
Source: JAMA Netw Open. 2022 Jun 9;5(6):e2216162. doi: 10.1001/jamanetworkopen.2022.16162 (PMC9185185; doi:10.1001/jamanetworkopen.2022.16162)
Supplement: Supplement 1. — Trial Protocol [file jamanetwopen-e2216162-s001.pdf]

Effect of Point-of-Care Testing for Respiratory Pathogens on Antibiotic Use in Children:

A Randomized Clinical Trial

Suvi Mattila, Niko Paalanne, Minna Honkila, Natalia Miettinen, Tytti Pokka, Terhi Tapiainen

TABLE OF CONTENT

	Pages
**Research Protocol**	**2-31**
**Statistical Analysis Plan**	**33-51**

THE CLINICAL IMPACT OF IMMEDIATE INDENTIFICATION OF RESPIRATORY PATHOGENS IN ACUTELY ILL CHILDREN: A RANDOMIZED CLINICAL TRIAL

HeVi Trial (Hengitystiepatogeenien Vieritestaus (Finnish), Point-of-care testing of respiratory pathogens)

TABLE OF CONTENTS

TABLE OF CONTENTS	Pages
Title page and table of contents	1
Final research protocol	2-19
Appendix 1. Educational guide for the interpretation of pathogen findings	20
List of amendments including dates	21-23
Literature review table of RCTs	24-32

THE CLINICAL IMPACT OF IMMEDIATE INDENTIFICATION OF RESPIRATORY PATHOGENS IN ACUTELY ILL CHILDREN: A RANDOMIZED CLINICAL TRIAL

HeVi Trial (Point-of-care testing of respiratory pathogens)

***Final* Study Protocol**

Ethics Committee: 8/2019

Suvi Mattila[1,2],	MD, Doctoral student, Doctor in training (Pediatrics)
Niko Paalanne[1,2],	MD, PhD, Specialist in pediatrics and pediatric infections
Minna Honkila[1,2],	MD, PhD, Specialist in pediatrics
Natalia Miettinen	MD
Tytti Pokka[2],	Biostatistician
Terhi Tapiainen[1,2]	Professor of Pediatrics

[1]Oulu University Hospital, Department of Children and Adolescents
[2]University of Oulu, PEDEGO research group

Contact information:
Terhi Tapiainen
Department of Pediatrics and Adolescent Medicine
Oulu University Hospital
PO Box 23
90029 Oulu
terhi.tapiainen@oulu.fi
Telephone: +358 40 70 546 77, +358 8 315 5185

1. ABSTRACT

Clinical problem

The field of microbiological diagnostics of respiratory infections has changed dramatically in recent years. There are now several **multiplex PCR devices** on the market which can provide an accurate diagnosis of the respiratory infection as early as **within one hour** of sampling. The test panels are able to analyze simultaneously about 20 respiratory pathogens. The multiplex PCR devices can be placed in the emergency room as they can also be used by acute care nurses. This means that the treating doctor can have access to the results during the emergency room visit.

Most pediatric infectious disease doctors consider active and rapid point-of-care (POC) diagnostics relevant because accurate diagnosis of the cause of respiratory infections could reduce the use of unnecessary antibiotics and hospitalizations and provide data to help with prognosis assessment. However, the role of diagnostics in improving patient care has been poorly demonstrated, and the equipment and tests are quite **expensive**. That is why **the clinical benefit of a rapid POC diagnostic devic**e placed in the pediatric emergency room **must be investigated** compared to current practice before the devices are widely adopted.

Study design

In this study, we investigate the effect of a new rapid POC testing device placed in the pediatric emergency room on the treatment of pediatric patients, **the onset of treatment with antibiotics and hospitalization rate** compared to current treatment, where the test is prescribed by the doctor and the result is interpreted according to standard practice in the hospital laboratory, and is usually available on the morning of the next working day.

2. CONCEPTS

Multiplex testing	Simultaneous analysis of several pathogens from one sample
Point-of-care (POC) testing	Diagnostic testing at or near the point of care, i.e. close to the patient
Pathogen	A disease-causing agent (viruses and bacteria)

3. BACKGROUND

3.1. Respiratory infections and antibiotic treatment in children

Respiratory infections with fever are a significant cause of pediatric morbidity and reason to seek emergency care. During the winter months, about 50% of all emergency room visits among children under 7 years are due to acute respiratory tract infections.[1] **Differentiating viral infections** from infections caused by bacteria in children with acute respiratory symptoms is a common clinical problem in the pediatric emergency room department.

Acute **respiratory infections in children are still a common reason for starting antibiotics**, even though the majority of respiratory tract infections in children are caused by viruses and do not require treatment with antibiotics. In a US study, treatment with antibiotics was started in 60% of children with pharyngitis although it is estimated that only 37% of the cases of pharyngitis in children are caused by bacteria.[2] In a Canadian study, 74% of the children attending emergency room due to respiratory infections were started on antibiotics; however, based on treatment guidelines, treatment with antibiotics was deemed unnecessary in half of the cases. [3]

The use of antibiotics increases individuals' risk of **antibiotic resistant bacterial strain carriage** and infections caused by these bacteria. Following the use of penicillin series antibiotics, the risk of antimicrobial-resistant pneumococcal carriage was fourfold, decreasing to 1.5-fold a month after the antibiotic therapy, however.[4] **The emergence of resistant strains is highest when the use of antibiotics is greatest.[5]** Children act as carriers of resistant bacterial strains and spread them to those around them. The aim of more targeted use of antimicrobials is to prevent the spread of resistant strains in the population and maintain the effect of antimicrobial agents.

Starting treatment with **macrolides** for lower respiratory tract infections is very common, even though the prevalence of atypical pathogens is low in Finnish data. The number of **mycoplasma** infections began to increase in 2010, and according to the Finnish National Infectious Diseases Register data, 52% and 14% of the cases were at that time diagnosed in patients aged 5 to 19 years and under 5 years of age, respectively.[6] In 2017, the number of laboratory-confirmed mycoplasma infections in Finland as a whole was 2,507. The widespread use of macrolides has contributed to the development of pneumococcal strains that are resistant to macrolides. With macrolide use, the risk

of a resistant pneumococcal strain nearly quadrupled, remaining 2- to 8-fold as long as three months after the end of the antibiotic treatment.[4]

Macrolides may also have **long-term effects** on children's gut microbiome.[7] In addition, the use of antibiotics may have other adverse effect on health besides the development of resistant strains. In particular, the use of wide-spectrum antibiotics in early childhood has also been linked to increased risk of **obesity**.[8, 9]

3.2 Previous studies on the effects of pediatric respiratory infection diagnostics

A systematic review (Doan et al. 2014)[10] of the effect of rapid POC testing for respiratory viruses on the antimicrobial treatment started, the need for additional testing and the length of emergency room stays showed that treatment with antibiotics was started less often in pediatric patients with a confirmed respiratory virus infection, but the difference between the groups was not statistically significant (RR 0.89, 95% CI 0.71; 1.12). The review included four studies (three randomized controlled studies and one group randomized study where the subjects were divided into groups according to day of the week); three of the studies focused on rapid influenza tests and one on simultaneous rapid testing for several respiratory viruses (Table 1, p. 4–5).

However, none of the studies assessed the effect of rapid (**one hour**) modern comprehensive POC multiplex testing on patients' care; instead, they **focused mainly on testing for influenza viruses** whereas respiratory viruses, such as **mycoplasma and pertussis, were not investigated** at all (**Table 1, p. 4–5**). Only one study used a multiplex test detecting several respiratory viruses[11], but the samples were analyzed in a laboratory and the results were only available during the laboratory office hours, so the test was not a genuine point-of-care test.

Study	Study design	Sample size	Age	Method	PATHOGENS	Results available (min)	OUTCOMES	Findings
**Doan et al. 2009**[11]	Single-center ROC	200	3–36 months	Rapid respiratory virus panel, direct immunofluorescence assay (SimulFluor respiratory screening agent)	Adenovirus, Influenza A and B, Parainfluenza 1, 2 and 3, RSV	30 - 150	Length of emergency room stay, need for further tests, antibiotics started	No significant difference in the number of antibiotics started (RR = 0.86, 95% CI = 0.48, 1.53) or other outcome measures.<br><br>Virus-positive subjects were prescribed fewer antibiotics if they sought treatment again within 7 days of the first visit (RR= 0.36; 95% CI = 0.14, 0.95)
**Iyer et al. 2006**[12]	Group-randomized controlled study	700	2 months –2 years	QuickVue Influenza test	Influenza	30	Laboratory, chest x-ray, use of antibiotics, cost of treatment period, length of stay, admittance to hospital, revisits	No significant difference between groups. Fewer antibiotics were prescribed for influenza-positive patients than for those who were influenza negative, but the POC test brought no added benefit. (15.7%, 95% CI 11.8, 19.5 vs. 16.6%, 95% CI 12.7, 20.5) OR 0.53 vs. 0.57, p = 0.703)
**Poehling et al. 2006**[13]	RCT	468	< 5 years	QuickVue Influenza test	Influenza		Diagnostic tests performed and antibiotics initiated	Fewer diagnostic tests were prescribed for the rapid test group (39% vs. 51%, *P* = .03). No difference in initiation of antibiotics (26% vs. 29% p = 0.75)
**Bonner et al. 2003**[14]	Single-center ROC	391	2 months –21 years	FluOIA (optical immunoassay)	Influenza	20-25	Need for further tests, antibiotics and antivirals, length of emergency room	Influenza-positive children aged 2–36 months whose influenza test result was know were prescribed fewer additional tests and antibiotics (4/52 vs. 23/70, p = 0.002). Reduced the number of antibiotics initiated RR = 0.66, 95%

							stay	CI 0.45; 0.96
**Ozkaya et al. 2009[15]**	Prospective case-control	97	3–14 years	Influenza A/B Rapid Test	Influenza	Not reported	Initiation of antibiotic	Fewer antibiotics were started for the group that underwent rapid influenza testing (32% vs. 100%, p <0.0001)
**Abanses et al. 2006[16]**	Prospective case-control (the planned group randomization did not take place)	1007	3 months – 3 years	Directigen Flu A+B	Influenza		Additional tests (lab and imaging), antibiotics initiated, cost of treatment, length of emergency room stay (min)	Significantly more RSV rapid tests (18% vs. 7%, RR 2.5. 95% CI 1.6-3.9) and chest x-rays (26% vs. 20%, RR = 1.3, 95% CI 1.01-1.7) were performed on those in the standard treatment group compared to those in the POD test group. There was no difference in the number of antibiotics initiated (30% vs. 35%, RR=0.84, 95% CI 0.70-1.02). Full blood counts (RR 12.0; 95% CI 2.9 – 49), blood cultures (RR = 12.0; 95% CI, 3.0 – 51.0), RSV tests (RR = 9.2; 95% CI, 3.4 – 25.0), urine samples (RR = 5.7; 95% CI, 2.0 – 16.0) and chest x-rays (RR, 2.2; 95% CI, 1.04–4.5) were more frequently taken from influenza-positive patients receiving standard treatment than from POC-tested influenza positive patients. In addition, the length of emergency room stays (195 vs. 156 min; 95% CI for the difference 19–60) was longer and the costs ($666 vs. $393; 95% CI for the difference 153–392) were higher.

**Esposito et al. 2003**[17]	Single-center ROC	957	0–15 years	QuickVue Influenza test	Influenza		Blood tests taken, chest x-rays, use of antibiotics, admittance to hospital	Those with a positive rapid test result were prescribed fewer blood tests (2.3% *vs.* 14.5% and 15.0%; $p = 0.045$ and $p = 0.038$) and antibiotics (32.6% *vs.* 64.8% and 61.8%; $p < 0.0001$ and $p = 0.0003$) than those with a negative rapid test results and those who had not been tested.

Table 1. Previous studies on the effect of respiratory virus diagnostics on children with acute respiratory infections

4. MATERIAL AND METHODS

4.1. Objective of the trial

The objective of the trial is to investigate whether immediate point-of-care testing of respiratory pathogens in the emergency room improves the treatment of acute pediatric patients compared to diagnostics prescribed by the doctor and conducted in the laboratory. In addition to reduction in antibiotics use, hospitalizations and revisits to the emergency room, the costs to the health care system are compared in the trial.

4.2 Trial design

The trial is a single-center, open, parallelly randomized controlled trial comparing immediate point-of-care testing for respiratory tract pathogens to a control group where samples are only taken at doctor's discretion and analyzed in a central laboratory during office hours on weekdays. As we assume that the trial group will benefit from the more rapid diagnostics enabled by the adoption of POC testing, the groups are randomized into two groups in a 2:1 ratio (POC testing : current practice).

4.3. *Sample and inclusion criteria*

The sample consists of children coming to the pediatric emergency room due to an acute respiratory tract infection or fever. During the day (8 am–midnight), acute pediatric patients requiring assessment in specialized health care are treated in the pediatric emergency room of Oulu University Hospital. During the night (midnight–8 am), pediatric patients from primary health care requiring emergency assessment are also treated in the emergency room.

The inclusion criterion for the trial is the presence of respiratory tract symptoms (at least one of the following: cough, rhinitis, increased respiratory rate, respiratory distress, sore throat or earache) and/or fever of at least 38.0°C, measured either in the emergency room or at home. There are no separate exclusion criteria for the trial in the emergency room, but no samples are taken from patients who are critically ill and require immediate resuscitation or similar treatment. Only patients whose family has given or who have themselves, together with their family, given a written consent to take part in the study are enrolled in the trial.

4.4. The randomized interventions compared:

i. Control group

Current treatment practice: ***The diagnostic tests needed are conducted in the laboratory during office hours following a clinical evaluation by the doctor who prescribed the test***

At the moment (since spring 2019), the following tests for respiratory tract pathogens are available in the emergency room of Oulu University Hospital. The tests are done in the laboratory and are prescribed by the on-call physician.

- Nucleic acid detection test for **influenza and RS virus**, analyzed in the laboratory; the results are available **approximately 2–3 hours** after sampling.
- In addition, since 2019, a test covering the most common **respiratory tract viruses as well as mycoplasma and pertussis**, which is done in the laboratory **during office hours** (Qiagen, Table 2**)**
- For both tests, the sample is most commonly taken from phlegm suctioned from the patient, or from the nasopharynx using a nylon or dacron swab.

ii. Point-of-care test group

The new treatment practice studied: ***On emergency room registration, a sample is taken from children with respiratory tract symptoms and/or fever. The sample is analyzed with the POC device in the emergency room, and the result is available during the emergency room visit.***

- The sampling technique does not differ from the current practice and the sample is taken from phlegm suctioned from the patient or from the nasopharynx using a nylon or dacron swab in children with runny noses, and in unclear cases (fever with no respiratory tract symptoms), from the pharynx.

4.5 Practical implementation of the trial

A POC device for the pediatric emergency room that is able to detect respiratory pathogens was put out to tender. The winner was Qiagen's Respiratory Panel 2, which detects the respiratory pathogens described in the table below. During the tendering process, the device was tested by the acute care nurses, who considered that it was usable in the emergency room setting.

Before initiation of the trial, the pediatric nurses were trained to use the device and clinicians were educated for interpretation of the results. The rough guideline for interpretation of pathogen findings was available for all attending physicians (Appendix 1).

Table 2. The respiratory pathogens detected by the POC device

Viruses	Bacteria
**Adenovirus**	***Bordetella pertussis***
**Bocavirus**	***Legionella pneumophila***
**Coronavirus 229E**	***Mycoplasma pneumoniae***
**Coronavirus HKU1**	
**Coronavirus NL63**	
**Coronavirus OC43**	
**human Metapneumovirus A/B**	
**Influenza A**	
**Influenza A subtype H1N1/2009**	
**Influenza A subtype H1**	
**Influenza A subtype H3**	
**Influenza B**	
**Parainfluenza virus 1**	
**Parainfluenza virus 2**	
**Parainfluenza virus 3**	
**Parainfluenza virus 4s**	
**Respiratory Syncytial virus A/B**	
**Rhinovirus/Enterovirus**	

Subjects are recruited for the trial by a pediatric emergency room nurse upon emergency room registration. The emergency room nurses have previous experience of recruiting patients from two clinical trials. The subjects recruited to the study can also discuss the trial with the on-call physician. The subjects are asked to provide a written consent, after which they are randomized into groups in a 2:1 ratio (treatment under study:current treatment). The interventions are randomized by trial numbers and enclosed in non-transparent envelopes. After asking for consent, the nurse opens the envelope assigned to the number that tells which group the subject is assigned to. Patients randomized to the new treatment practice are asked to give a sample which is analyzed with the POC device in the emergency room. If there are technical problems with the analysis, the nurse may take a new sample if necessary; a note on the technical problem is entered in the device log. During the trial, the number of technical problems with analysis increased, and the device manufacturer was informed of all tests that had failed. During the trial, over a short period from Sep 8 to 28, 2019, an increase in the number of failed samples was observed (9 out of 56, i.e. 16% of all samples analyzed). According to the manufacturer, this was to a significant degree (4 out 9, 45%) associated with problems with the sample cassette transportation chain and wrong storage temperature during transportation. As technical problems persisted, the operating system of the POC test device was updated on Jan 7, 2020.

If an unforeseen adverse event occurs during sampling, an adverse event report is recorded in the hospital's internal adverse event reporting system; in addition, the investigators are informed of the event. The POC test results are automatically recorded in Weblab and are thus available at the doctor's appointment. Samples are only taken at the on-call physician's discretion from the children randomized to the current treatment group. The samples are stored in the emergency room and transported to the laboratory for analysis at 7 am the next morning. The results are entered into Weblab in the microbiology lab, and the on-call physician is responsible for reporting the results and reacting to them.
Due to the SARS-CoV2 epidemic and change in testing practices, randomization was halted on March 13, 2020 at 3 pm when 1,350 subjects had been recruited.

4.6. Outcome measures

Primary outcome measure

- Proportion of children with antibiotic prescription at emergency room

Secondary outcome measures

- Treatment with antibiotics initiated within a week (7 days) of the emergency room visit and different groups of antibiotics
- Macrolide antibiotics prescribed in the ED
- Macrolide antibiotics started in infants under 3 months (proportion)
- Immediate admittance to hospital from emergency room (hospitalized pediatric patients)
- Pediatric patients admitted to hospital within a week (7 days) of the emergency room visit
- Diagnostic tests performed in the emergency room
- Outpatient phone contacts and their number (with nurse or physician) within 7 days
- Revisits to emergency room within the next 7 days
- Ancillary laboratory testing
- Intensive care or intensive monitoring within the next 30 days after the emergency room visit
- Mortality within the next 30 days after the emergency room visit
- Visit associated costs
- Length of stay at ED
- Pathogen directed therapy (defined as antimicrobial therapy directed against detected pathogen with specific treatment available)
- Time from emergency room visit to onset of pathogen-directed treatment

4.7. Sample size

The annual number of visits to the pediatric emergency room at Oulu University Hospital is about 4,000–5,000; of these, about 50–70% are related to infections. The main outcome measure in the study is the number of antibiotic treatment courses initiated. Before the study in 2015, in a sample of 1195 children treated at the pediatric ED, antibiotics were administered to 31% (95% confidence interval [CI] 28%-34%) of the patients. Since we assumed that approximately every third child (33%) of the children in the control group would receive antibiotics and considered a relative reduction of 25% to be clinically significant, we estimated that, with an alpha error of 5% and a statistical power of 80% and using 2:1 randomization, we needed 785 case subjects and 392 controls, i.e. a total of 1177 children. We planned to recruit participants for one epidemiological

year, from May 2019 to May 2020. We considered a 25% reduction in antibiotic therapy to be clinically significant, as was done in the review of Doan et al. (2014). Based on the calculation, the sample size reached was considered sufficient in terms of statistical power even if the randomization had to be stopped prematurely due to Covid-19 pandemic.

4.8. Randomization

Because the study is a single-center open trial, randomization is done in 2:1 permuted blocks, the size of which varies randomly between 3, 6, and 9. The randomization is done using computer-generated random sequence numbers by a biostatistician not involved in data gathering. The biostatistician draws up a list of numbers. After this, a study nurse who is not involved in the trial or data gathering places the interventions in non-transparent envelopes with a running trial number (1-1668). After randomization, the study is an open study, because the outcome measures compared are not subjective but data collected from medical records, and randomization would not be relevant in this setting.

The randomization of patients was stopped on Mar 13, 2020 at 3 pm due to the SARS-CoV2 pandemic.

4.9. Statistical methods

The subjects are analyzed in the groups (*intention to treat*) they were randomized into. In the case of failure to comply with the study protocol, a secondary analysis is performed where the subjects are grouped according to the implemented intervention (*per protocol*). The proportion of children with an antibiotic prescription in the ED is reported separately for untargeted and pathogen-targeted antibiotic therapy.

If the POC test result is available for less that 70% of the patients randomized to the rapid group at the moment of decision-making, a separate per protocol analysis is done on the patients for whom the test result was available.

The statistical significance of the primary outcome measure is analyzed with SND (standard normal deviation) test. The difference between proportions is compared using the SND test, and 95% CI is reported for the differences between the proportions. Continuous outcomes (length of emergency

room stay, difference between cost of treatment) is analyzed using t test and 95% CI is reported. An alpha-error level of 5% (0.05) is used as cut-off value for P. New emergency room visits and hospital admissions are analyzed with Kaplan-Meier method using time-to-event analysis.

Of group demographic variables, means and standard deviations are reported when describing the patient material. As the differences between the groups are coincidental they are not tested statistically.

The Study Design section is written using the CONSORT 2010 checklist. The Ethics Committee of the Northern Ostrobothnia Hospital District gave a favorable opinion on the study protocol on Mar 20, 2019. On Apr 1, 2020, the Northern Ostrobothnia Hospital District granted permission for the trial comparing treatment methods. The trial was registered in clinicaltrials.gov on Apr 18, 2019. Recruitment of subjects commenced on May 6, 2019. Randomization was discontinued on Mar 13, 2020 due to the pandemic.

5. ETHICAL CONSIDERATIONS

To our knowledge, a comprehensive POC test for respiratory tract pathogens is currently not in use in Finnish pediatric clinics and there are no current care guidelines on its use. Intuitively, one might think that more rapid and comprehensive diagnostics would definitely bring benefits to patient care in the form of more accurate diagnostics and shorter treatment times. However, the benefits of routine testing for respiratory pathogens in the emergency room have not been demonstrated in a randomized controlled setting. The benefit of testing must be scientifically evaluated in a university clinic before adoption into routine use.

Pediatric patients seeking emergency treatment for fever and/or respiratory symptoms are recruited to the trial. Because the aim is to achieve a representative data set, no exclusion criteria are determined. The test result is interpreted by the on-call physician; if necessary, after consulting the back-up on-call physician. Patients in very poor condition who require immediate intensive care and whose status does not allow any unnecessary invasive procedures are not recruited to the trial. During the study, the POC device is used for research purposes only; it is not used for emergency room patients who have not been recruited to the study. The study is limited to the emergency room. However, outside the emergency room, doctors in the ICU or hematology ward can order a

POC test on a patient on these wards if they consider that it would be of significant benefit in their care.

Samples for the trial are collected in the same way as in current routine practice. The acute care nurses have a lot of experience of taking samples. The samples taken for the trial are used for diagnosis and for making decisions on treatment during the emergency room visit.

Participation in the trial is voluntary. Children under 6 years of age are recruited with consent from a parent. Pediatric patients older than 6 years who are recruited to the trial are given age-appropriate information about the study and they are asked to provide a separate consent, after which consent is also requested from a parent/guardian. Adolescents older than 15 years can themselves decide whether they want to take part in the trial, and if they do, their parents are sent information about their child's participation in the trial. The subject, parent or guardian has the right to ask additional questions and discuss the trial with the on-call physician, who can call the trial doctors if necessary. Consent that has already been given can be withdrawn at any stage. Pediatric patients who refuse to take part in the trial are treated in the emergency room according to current normal practice and the refusal has no effect on the child's future care. The trial has been granted a favorable opinion by the Ethics Committee of Northern Ostrobothnia Hospital District and a description of the study register will be drawn up. All trial information will be handled confidentially and a separate assessment of privacy risk concerning the study data will be done. The patients' and controls' data is stored in the hospital's data system protected by a user ID and password. The study forms (consent forms) are stored in a locked space in the Department of Children and Adolescents, Oulu University Hospital (room L6 219). The data are analyzed in anonymized form (with no personal identifiers) after pooling of all clinical data.

6. CLINICAL RELEVANCE OF THE TRIAL

Antimicrobial resistance is a growing problem that may in the future endanger the treatment of infections which are currently treatable. To combat antimicrobial resistance, the WHO has published an action plan where one of the strategic aims is optimization of antimicrobial use. Children act as carriers of respiratory pathogens, and as resistant bacterial strains become more common, they also spread them efficiently. Rapid, targeted diagnostics of respiratory pathogens

may help target the use of antimicrobials more precisely and reduce the growth of antibiotic resistance.

The spectrum of microbes that cause acute respiratory infections is wide, and clinically, it is often difficult to tell the difference between bacterial and viral symptoms. However, in terms of treatment, it is crucial to know whether the illness is caused by a virus or bacteria. POC testing of respiratory pathogens sheds more light on the matter. However, the result of a POC test based on nucleic acid detection does not necessarily indicate the presence of a live organism; instead of an infection that can be treated, the finding may be due to colonization, infection with no symptoms, reactivation or prolonged virus shedding, which is why clinical discretion is required when interpreting the results of POC tests. The use of POC testing causes additional costs, but if its use helps reduce the need for additional tests or monitoring in hospital, the total cost of treatment may be lower. A precondition for the rational use of POC tests is that they have an impact on treatment decisions and save cost, and do not put a strain on healthcare resources.

The aim of this trial is to find an additional means of targeting antimicrobial therapy and to demonstrate the clinical benefit and cost-effectiveness of a new microbiological diagnostic test before its more widespread adoption.

The trial also provides additional information about the utilization of POC diagnostics during a pandemic, the prevalence of SARS-CoV2 virus in children, and the need of hospital treatment among pediatric patients requiring assessment in specialized care.

References

1. Bourgeois FT, Valim C, Wei JC, McAdam AJ, Mandl KD. Influenza and other respiratory virus-related emergency department visits among young children. *Pediatrics*. Jul 2006;118(1):e1-8. doi:10.1542/peds.2005-2248
2. Dooling KL, Shapiro DJ, Van Beneden C, Hersh AL, Hicks LA. Overprescribing and inappropriate antibiotic selection for children with pharyngitis in the United States, 1997-2010. *JAMA Pediatr*. Nov 2014;168(11):1073-4. doi:10.1001/jamapediatrics.2014.1582
3. Wang EE, Einarson TR, Kellner JD, Conly JM. Antibiotic prescribing for Canadian preschool children: evidence of overprescribing for viral respiratory infections. *Clin Infect Dis*. Jul 1999;29(1):155-60. doi:10.1086/520145
4. Bakhit M, Hoffmann T, Scott AM, Beller E, Rathbone J, Del Mar C. Resistance decay in individuals after antibiotic exposure in primary care: a systematic review and meta-analysis. *BMC Med*. Aug 7 2018;16(1):126. doi:10.1186/s12916-018-1109-4
5. Bell BG, Schellevis F, Stobberingh E, Goossens H, Pringle M. A systematic review and meta-analysis of the effects of antibiotic consumption on antibiotic resistance. *BMC Infect Dis*. Jan 9 2014;14:13. doi:10.1186/1471-2334-14-13
6. Puolakkainen M. JA. Mycoplasma pneumoniae -infektiot. *Duodecim*. 2012;128(21):2236-43.
7. Korpela K, Salonen A, Virta LJ, et al. Intestinal microbiome is related to lifetime antibiotic use in Finnish pre-school children. *Nat Commun*. Jan 26 2016;7:10410. doi:10.1038/ncomms10410
8. Bailey LC, Forrest CB, Zhang P, Richards TM, Livshits A, DeRusso PA. Association of antibiotics in infancy with early childhood obesity. *JAMA Pediatr*. Nov 2014;168(11):1063-9. doi:10.1001/jamapediatrics.2014.1539
9. Saari A, Virta LJ, Sankilampi U, Dunkel L, Saxen H. Antibiotic exposure in infancy and risk of being overweight in the first 24 months of life. *Pediatrics*. Apr 2015;135(4):617-26. doi:10.1542/peds.2014-3407
10. Doan Q, Enarson P, Kissoon N, Klassen TP, Johnson DW. Rapid viral diagnosis for acute febrile respiratory illness in children in the Emergency Department. *Cochrane Database Syst Rev*. Sep 15 2014;(9):CD006452. doi:10.1002/14651858.CD006452.pub4
11. Doan QH, Kissoon N, Dobson S, et al. A randomized, controlled trial of the impact of early and rapid diagnosis of viral infections in children brought to an emergency department with febrile respiratory tract illnesses. *J Pediatr*. Jan 2009;154(1):91-5. doi:10.1016/j.jpeds.2008.07.043
12. Iyer SB, Gerber MA, Pomerantz WJ, Mortensen JE, Ruddy RM. Effect of point-of-care influenza testing on management of febrile children. *Acad Emerg Med*. Dec 2006;13(12):1259-68. doi:10.1197/j.aem.2006.07.026
13. Poehling KA, Zhu Y, Tang YW, Edwards K. Accuracy and impact of a point-of-care rapid influenza test in young children with respiratory illnesses. *Arch Pediatr Adolesc Med*. Jul 2006;160(7):713-8. doi:10.1001/archpedi.160.7.713
14. Bonner AB, Monroe KW, Talley LI, Klasner AE, Kimberlin DW. Impact of the rapid diagnosis of influenza on physician decision-making and patient management in the pediatric emergency department: results of a randomized, prospective, controlled trial. *Pediatrics*. Aug 2003;112(2):363-7. doi:10.1542/peds.112.2.363
15. Ozkaya E, Cambaz N, Coskun Y, Mete F, Geyik M, Samanci N. The effect of rapid diagnostic testing for influenza on the reduction of antibiotic use in paediatric emergency department. *Acta Paediatr*. Oct 2009;98(10):1589-92. doi:10.1111/j.1651-2227.2009.01384.x
16. Abanses JC, Dowd MD, Simon SD, Sharma V. Impact of rapid influenza testing at triage on management of febrile infants and young children. *Pediatr Emerg Care*. Mar 2006;22(3):145-9. doi:10.1097/01.pec.0000202454.19237.b0
17. Esposito S, Marchisio P, Morelli P, Crovari P, Principi N. Effect of a rapid influenza diagnosis. *Arch Dis Child*. Jun 2003;88(6):525-6. doi:10.1136/adc.88.6.525

Appendix 1. Educational guide for the interpretation of pathogen findings available in the emergency department.

Positive findings	Negative findings
• **Influenzavirus**<br>➔ Start oseltamivir<br>• ***Mycoplasma pneumoniae***<br>➔ >Start macrolide antibiotic<br>• ***Bordetella pertussis***<br>➔ Start macrolide antibiotic<br>• **Adenovirus with tonsillitis and high c-reactive protein**<br>➔ Generally antibiotics are not useful<br>• **Human metapneumovirus, Parainfluenza virus, Coronavirus, RSV**<br>➔ Supports the clinical diagnosis of a viral infection<br>• **Bocavirus**<br>➔ Consider course of illness: wheezing mainly indicates viral etiology<br>• **Picornavirus (Enterovirus or Rhinovirus)**<br>➔ Consider investigating enterovirus PCR if infant and severe course of illness<br>➔ Consider c-reactive protein and possible coinfection with *S. pneumoniae*	• ***Mycoplasma pneumoniae***<br>➔ Usually no macrolide antibiotics<br>• ***Bordetella pertussis***<br>➔ Usually no macrolide antibiotic<br><br>**For all pathogens:** Consider the general condition of the patient and the level of c-reactive protein in the clinical decision making

Attachment 1: Amendments to the study protocol

Version 2, Mar 27, 2020

- Section 4.5 *Practical implementation of the trial:* the POC device (Qiagen) selected after tendering has been added, the comparison table has been deleted, and a table of the pathogens detected by the POC device has been added
- Macrolides started for infants under 3 months have been added to outcome measures
- Addition to Section 4.7. *Sample size*: "If the targeted sample size is reached before 12 months, data gathering will continue for a total of 12 months to achieve a sample that covers a whole epidemiological year. "
- A reference to the favorable opinion issued by the Ethics Committee has been added to the study protocol

Version 3, Apr 18, 2019

- Outcome measures (section 4.6.) have been amended as follows: Macrolide antibiotics started in infants under 3 months (proportion)
- The following additions and clarifications have been made to Section 4.5:
  - A note on whether the patient belongs to rapid test or POC test group is recorded in Hoitu
  - Results of the POC test are recorded automatically in Weblab
  - The samples taken from controls are stored in the emergency room and transported to the laboratory for analysis at 7 am the next morning.
- The trial was registered in the ClinicalTrials database on Apr 18, 2019.
- Recruitment of subjects starts on May 6, 2019.

Version 4, May 15, 2019

- The following addition has been made to Section 4.5: "If there are technical problems with the analysis, the nurse may take a new sample if necessary; a note on the technical problem is entered in the device log. "

- Reporting of adverse effects is described in more detail in Section 4.5 "If an unforeseen adverse event occurs during sampling, an adverse event report is recorded in the hospital's internal adverse event reporting system; in addition, the investigators are informed of the event."
- The targeted sample size is described in more detail in Section 4.7 Sample size: "Some patients revisit the emergency room and they can be recruited again if the clinical picture has deteriorated significantly or a new illness is suspected, but only first visits are included in the targeted sample size. "

Version 5, Dec 19, 2019

- Investigation and reporting of technical problems that appear during the trial is described in more detail in Section 4.5: "During the trial, the number of technical problems with analysis increased, and the device manufacturer was informed of all tests that had failed. During the trial, over a short period from Sep 8 to 28, 2019, an increase in the number of failed samples was observed (9 out of 56, i.e. 16% of all samples analyzed). According to the manufacturer, this was to a significant degree (4 out 9, 45%) associated with problems with the sample cassette transportation chain and wrong storage temperature during transportation. As technical problems persisted, the operating system of the POC test device was updated on Jan 7, 2020."
- The following outcome measures have been added to secondary outcome measures:
    - Antiviral therapies started at emergency room visit
    - Antiviral therapies started within 7 days after the emergency room visit

Version 6, Feb 12, 2020

- The following have been added to outcome measures:
    - Time from emergency room visit to initiation of pathogen-directed treatment

- The following has been added to statistical methods: "If the POC test result is available for less that 70% of the patients randomized to the rapid group at the moment of decision-making, a separate per protocol analysis is done on the patients for whom the test result was available. "

Version 7, Feb 18, 2020

Assessment of required sample size is described in more detail as follows:

Due to Covid-19 pandemic, in mid-February 2020, we re-evaluated the statistical power to decide whether we will complete or discontinue the study if the Covid-19 pandemic will make the recruitment impossible. Based on the calculation, the sample size reached was considered sufficient in terms of statistical power even if the randomization had to be stopped prematurely due to Covid-19 pandemic.

Version 8, Mar 16, 2020

- A reference to termination of randomization was added to Section 4.8: The randomization of patients was stopped on Mar 13, 2020 at 3 pm due to the SARS-CoV2 pandemic.

Version 9, Jan 7, 2021

- **<u>A summary tables of full updated literature review</u>** are attached

Version 10, Sep 7, 2021

Addition to *4.9. Statistical methods:*
"The proportion of children with an antibiotic prescription in the ED is reported separately for untargeted and pathogen-targeted antibiotic therapy."

Version 11, Mar 29, 2022

Addition to *4.6. Outcome measures*
The definition for "Pathogen directed therapy" has been specified as follows:
"defined as antimicrobial therapy directed against detected pathogen with specific treatment available"

Table 1. Studies on clinical impact of point-of-care diagnostics of respiratory pathogens in acutely ill children with respiratory symptoms: study design, sample sizes, methods and tested pathogens.

Study	Study design	Sample size	Age	Method	Pathogens tested	Results available (min)	Results available prior to ED visit
**Bonner et al.[1] 2003**	Single center RCT	391	2 months to 21 years<br><br>Data analysed separately in age group 2 to 36 months with similar results	**Antigen test**<br><br>FluOIA (optical immunoassay)	Influenza	20 - 25	+
**Esposito et al.[2] 2003**	Single center RCT	957	0 to 15 years	**Antigen test**<br><br>(QuickVue Influenza test)	Influenza	10	+
**Abanses et al.[3] 2006**	Prospective<br><br>case-control<br><br>Randomization failed and the data was analysed as a convenience sample	1007	3 months to 3 years	**Antigen test**<br><br>Directigen Flu A+B	Influenza	**--**	+
**Iyer et al.[4]**	Quasi-randomized	700	2 months to 2	**Antigen test** (QuickVue Influenza	Influenza	30	+

**2006**	controlled study<br><br>(the method of testing was alternated by day)		years	test)		Up to 30 minutes	
**Poehling et al.[5] 2006**	Cluster randomized<br><br>RCT<br><br>(study days were randomized to rapid test or no rapid test days)	468	Up to 5 years	**Antigen test**<br><br>(QuickVue Influenza test)<br><br>(All participants in both groups were tested with viral culture and PCR as the criterion standard)	Influenza	Not reported	+
**Doan et al.[6]**<br><br>**2009**	Single center RCT	200	3 to 36 months	Rapid respiratory virus panel, **direct immunofluorescence assay** (SimulFluor respiratory screening agent)	Adenovirus, Influenza A and B, Parainfluenza 1, 2 and 3, RSV	30 - 150<br><br>+ rapid test available only during laboratory hours (8 am to 9 pm during weekdays and 8 am to 6:30 pm during weekends)	+
**Wishaupt et al.[7] 2011**	a controlled clinical trial,<br><br>Samples were randomized in the laboratory: samples with even order numbers were	583	Up to 12 years	RT-PCR	Intervention:<br><br>RSV A, RSV B, influenza viruses A and B, adenovirus, parainfluenza viruses 1, 2, 3, and 4, human bocavirus, coronaviruses 229E, OC43, and NL63, human metapneumovirus, rhinovirus, *Chlamydophila pneumoniae*, and *Mycoplasma*	Following day	Results reported on the following day

	assigned to intervention and samples with odd numbers were assigned to control group				*pneumonia, Bordetella pertussis*<br><br>*All patients;* **DFA**s for RSV, influenza A and B, adenovirus, and parainfluenza viruses 1, 2, and 3 and viral culture		
**Schechter-Perkins et al.[8] 2019**	Single center randomized controlled trial	197	4 months to 82 years	multiplex real time Rt-PCR assay<br><br>(cobas® Liat Influenza A/B device)	Influenza A/B	20 minutes<br><br>Subjects enrolled Monday to Friday, 8 am to 11 pm	+
**Reichl et al.[9]**<br><br>**2020**	Retrospective observational study of children admitted to the infectious disease ward with retrospective cohort as a control group	786	Up to 16 years	RT-PCR<br><br>(FA Respiratory panel)	adenovirus, coronavirus 229E, coronavirus HKU1, coronavirus NL63, coronavirus OC43, human metapneumovirus, influenza A, influenza A subtype H1, influenza A subtype H3, influenza A subtype H1-2009, influenza B, parainfluenza virus 1, parainfluenza virus 2, parainfluenza virus 3, parainfluenza virus 4, human rhinovirus/enterovirus, respiratory syncytial virus, *Bordetella pertussis*, *Chlamydophila pneumoniae,* and *Mycoplasma pneumoniae*	Two working days	-

578 **Table 2. Studies on clinical impact of point-of-care diagnostics of respiratory pathogens in acutely ill children with respiratory symptoms: outcomes**

Study	Antibiotic prescription rate	Antiviral prescription rate	Hospital admission rate	Length of ED visit	Readmission to ED	Rate of ancillary tests	Cost efficiency
**Bonner et al.[1] 2003**<br><br>Data analysed separately in age group 2 to 36 months with similar results	*Influenza positive MD Aware (n=96) vs MD unaware (n=106)*<br><br>**7/26 vs 26/106 p=< 0.001**<br><br>*Influenza negative MD aware (n=97) vs MD unaware (N=92)*<br><br>27/97 vs 27/92<br><br>p=0.818	*Influenza positive MD Aware (n=96) vs MD unaware (n=106)*<br><br>**18 vs 7, p = 0.02**<br><br>*Influenza negative MD aware (n=97) vs MD unaware (N=92)*<br><br>0 vs 2, p=0.236	Not reported	*Influenza positive MD Aware (n=96) vs MD unaware (n=106)*<br><br>**25 vs 49 min (mean time from examination to discharge)**<br><br>**p=<0.001**<br><br>*Influenza negative MD aware (n=97) vs MD unaware (N=92)*<br><br>45 vs 42 min, p=0.549	Not reported	*Influenza positive MD Aware (n=96) vs MD unaware (n=106)*<br><br>**CBC 0 vs 13, p<0.001**<br><br>**Blood culture 0 vs 11, p<0.001**<br><br>**Urinanalysis 2 vs 12, p=0.011**<br><br>**Urine culture 3 vs 14, p= 0.011**<br><br>Cerebrospinal fluid study/cultre<br><br>0 vs 2, p= 0.499<br><br>**Chest radiograph 7 vs 26, p=0.001**<br><br>*Influenza negative MD aware (n=97) vs MD unaware (N=92)*<br><br>CBC 13 vs 7, p=0.196<br><br>Blood culture 12 vs 6, p=0.172<br><br>Unrinalalysis 10 vs 8, p=0.706<br><br>Urine culture 12 vs 5 p=0.096<br><br>Cerebrospinal fluid	*Influenza positive MD Aware (n=96) vs MD unaware (n=106)*<br><br>(mean charge/patient, laboratory and radiograph)<br><br>**15.65 $ vs 92.37 $, p<0.001**<br><br>*Influenza negative MD aware (n=97) vs MD unaware (N=92)*<br><br>93.07 $ vs 68.91 $, p=0.871

						study/cultre<br>3 vs 2, p= 0.695<br>Chest radiograph 22 vs 23, p=0.708	
**Esposito et al.[2] 2003**<br><br>At the time of the study, no antiviral drug was approved for use in the therapy of influenza in children	*Influenza positive (n=43) vs influenza negative (n=435)*<br><br>**32.6% vs 64.8% p<0.0001**<br><br>*Influenza positive (n=43)vs no test (n=479)*<br><br>**32.6% vs 61.8% p=0.0003**	*Influenza positive (n=43) vs influenza negative (n=435)*<br><br>0 vs 0 %<br><br>*Influenza positive (n=43)vs no test (n=479)*<br><br>0 vs 0%	*Influenza positive (n=43) vs influenza negative (n=435)*<br><br>0 vs 4.6 % p=0.240<br><br>*Influenza positive (n=43)vs no test (n=479)*<br><br>0 vs 5.8 % p= 0.154	Not reported	Not reported	*Influenza positive (n=43) vs influenza negative (n=435)*<br><br>**Blood examination**<br>**2.3 % vs 14.5 %, p=0.045**<br>Chest radiograph<br>4.6 % vs 11.7 %, p=0.207<br><br>*Influenza positive (n=43)vs no test (n=479)*<br>**Blood examination**<br>**2.3 % vs 15.0 % p=0.038**<br>Chest radiograph<br>4.6 % vs 11.7 % p=0.208	Not reported
**Abanses et al.[3] 2006**<br><br>Randomization failed and the data was analysed as a convenience sample	Standard protocol (n=719) vs tested in triage (n=288)<br><br>30 % vs 35 %,<br>RR 0.84 (0.70-1.02)	Not reported	Not reported	Standard protocol (n=719) vs tested in triage (n=288)<br><br>185+/- 80 min vs 185 +/-86, (CI for mean difference	Not reported	Standard protocol (n=719) vs tested in triage (n=288)<br><br>CBC 22 vs 17 % RR 1.3 (95% CI 0.95-1.7)<br>BC 21 % vs 17 % RR 1.2 (95% CI 0.95-1.7)<br>RSV testing 18 % vs 7.3 %,	Standard protocol (n=719) vs tested in triage (n=288)<br><br>total charges ($)<br>544 +/- 358 vs 538 +/- 427, CI for mean difference

				-12 -12)		RR 2.5 (1.6-3.9)<br>UA 18 % vs 14 %, RR 1.4 (0.98-1.9)<br>LP 0.3% vs 0%<br>**CXR 26 % vs 20 %, RR 1.3 (1.01-1.7)**	
**Iyer et al.**[4]<br>**2006**	Point of care (n=345) vs standard test (n = 355), % (95% CI)<br>Antibiotics given in ER<br>15.7 % (11.8, 19.5) vs 16.6 % (12.7, 20.5)<br>Discharged with antibiotic prescription<br>25.3 % (20.3, 30.2) vs 30.5 % (25.4, 35.6)	Not reported	11.6 % (8.2, 15.0) vs 10.4 % (7.2, 13.6)	203.6 min (194.4, 212.9) vs 204.1 min (194.5, 213.8)	Revisit to ED within 14 days<br>17.7 % (13.6, 21.7) vs 15.8 % (12.0, 19.6)	Blood culture 24.1 % (19.5, 28.6) 27.9 % (23.2, 32.6)<br>CBC 25.5 % (20.9, 30.1) vs 29.3 % (24.5, 34.1)<br>Urine culture 20.3 % (16.0, 24.6) vs 20.9 % (16.6, 25.1)<br>Lumbar puncture 2.0 % (0.5, 3.5) vs 1.4 % (0.2, 2.6)<br>Chest radiograph 24.9 (20.3, 29.5) vs 28.2 % (23.5, 32.9)<br>Chest radiograph 86 24.9 (20.3, 29.5) 100 28.2 (23.5, 32.9	625 $ (566, 692)<br>vs 592 $ (540, 650)
**Poehling et al.**[5]<br>**2006**	Emergency department<br>Rapid test (n=135) vs no rapid test	Emergency department<br>Rapid test (n=135) vs no rapid test	Not reported	Not reported	Not reported	Emergency department<br>Rapid test (n=135) vs no rapid test (n=170)	Not reported

	(n=170)<br>39 % vs 52 %, p=0.03<br>32 % vs 29 %, p=0.57<br>Acute care clinic Rapid test (n=70 vs No Rapid Test (n=93)<br>26 % vs 29 %, p=0.75	(n=170)<br>39 % vs 52 %, p=0.03<br>1 % vs 0 p=0.44<br>Acute care clinic Rapid test (n=70 vs No Rapid Test (n=93)<br>0 vs 0 %				Any diagnostic test<br>39 % vs 52 %, p=0.03<br>Chest radiograph 23 % vs 33 %, p=0.06<br>Blood count/culture 10 % vs 18% p= 0.05<br>Urinanalysis 13 % vs 16%, p=0.53<br>Acute care clinic Rapid test (n=70 vs No Rapid Test (n=93)<br>Any diagnostic test<br>17 % vs 13 %, p=0.45<br>Chest radiograph 9 % vs 5 %, p=0.42<br>Blood count/culture 4 % vs 3 % p= 1.00<br>Urinanalysis 7 % vs 3 %, p=0.29	
**Doan et al.[6]**<br>**2009**	VIRAP (n=89) vs control (n=110)<br>18 % vs 20.9 % RR 0.86, 95% CI (0.48, 1.53)<br>**Post ED Antibiotic**	Not reported	Not reported	105.7 (188.04) min vs 156.1 (235.82) min, mean difference -50.4 (-104.6, 3.7)	Within 7 to 10 days<br>33.7 % vs 39.1 %, RR 0.86 (0.59-1.25)	Chest X-ray 23.6 % vs 33.6 %, RR 0.70 (0.44, 1.11)<br>Blood work<br>10.1 % vs 17.3 %, RR 0.59 (0.28, 1.23)<br>Urine analysis 31.5 % vs 28.2 %, RR 1.12 (0.73-1.71)	Not reported

	**within 7 to 10 days 5.6 % vs 15.5 % RR 0.36 (0.14, 0.95)**					Post ED ancillary test<br><br>1.1 % vs 5.5 %, RR 0.21 (0.03, 1.7)	
**Wishaupt et al.[7] 2011**	intervention (n=298) vs control (n=285)<br><br>**41.6 % vs 27.4 % p=0.000**	74.8 % vs 74 % p=0.825	Not reported	time in hospital (days)<br><br>3.68 +/-2.68 vs 3.96+/-2.67 p=0.170	Not reported	Not reported	Not reported
**Schechter-Perkins et al.[8] 2019**	core lab (n=97) vs point of care (n=100)<br><br>14.4% vs 14.0 %, p=0.93	Not reported	Not reported	185.9 (110.0) vs 168.9 (91.7), p=0.26	Not reported	Not reported	Not reported
**Reichl et al.[9]**<br><br>**2020**	Study group (n=322) vs control group (n=464)<br><br>45 % vs 42.5 %<br><br>p= 0.784	1.9 % vs 0.9 %	Not reported	Length of stay at hospital (days)<br><br>mean 4.7 +/- 5.4 vs 4.7 +/- 4.4	Not reported	Not reported	Not reported

References

1.**Bonner AB**, Monroe KW, Talley LI, Klasner AE, Kimberlin DW. Impact of the rapid diagnosis of influenza on physician decision-making and patient management in the pediatric emergency department: results of a randomized, prospective, controlled trial. Pediatrics. 2003 Aug;112(2):363-7

2. **Esposito S**, Marchisio P, Morelli P*, et al*. Effect of a rapid influenza diagnosis. *Archives of Disease in Childhood* 2003;**88:**525-526.

3. **Abanses**, J. C., Dowd, M. D., Simon, S. D., and Sharma, V. (2006). Impact of rapid influenza testing at triage on management of febrile infants and young children. *Pediatr. Emerg. Care* 22, 145–149

4. **Iyer SB**, Gerber MA, Pomerantz WJ, Mortensen JE, Ruddy RM. Effect of point-of-care influenza testing on management of febrile children. *Academic Emergency Medicine* 2006;13(12):1259-68.

5. **Poehling KA**, Zhu Y, Tang YW, Edwards K. Accuracy and impact of a point-of-care rapid influenza test in young children with respiratory illnesses. *Archives of Pediatrics and Adolescent Medicine* 2006;160(7):713-8

6. **Doan Q**, Kissoon N, Whitehouse S, Dobson S, Cochrane D, Schmidt B, et al. A randomized, controlled trial of the impact of early and rapid diagnosis of viral infections in children brought to an Emergency Department with febrile respiratory tract illnesses. *Journal of Pediatrics* 2009;154(1):91-5.

7. **Wishaupt JO**, Russcher A, Smeets LC, Versteegh FG, Hartwig NG. Clinical impact of RT-PCR for pediatric acute respiratory infections: a controlled clinical trial. *Pediatrics*. 2011;128(5):e1113-e1120

8. **Schechter-Perkins EM**, Mitchell PM, Nelson KP, Liu JH, Shannon A, Ahern J, Orr B, Miller NS. Point-of-care influenza testing does not significantly shorten time to disposition among patients with an influenza-like illness. Am J Emerg Med. 2019 May;37(5):873-878

9. **Reischl**, AT, Schreiner, D, Poplawska, K, et al. The clinical impact of PCR-based point-of-care diagnostic in respiratory tract infections in children. *J Clin Lab Anal*. 2020; 34:e23203

THE CLINICAL IMPACT OF IMMEDIATE INDENTIFICATION OF RESPIRATORY PATHOGENS IN ACUTELY ILL CHILDREN:

A RANDOMIZED CLINICAL TRIAL

HeVi Trial (Point-of-care testing of respiratory pathogens)

<u>Final</u> Statistical Analysis Plan

PROTOCOL: Hevi Trial Study Protocol Version 11.0

VERSION: Version 7.0

DATE FINAL: Mar 29th 2022

APPROVAL PAGE

This document was reviewed by the following HeVi team members:

Suvi Mattila[1,2], MD, Doctoral student, Doctor in training (Pediatrics)

Niko Paalanne[1,2], MD, PhD, Specialist in pediatrics and pediatric infections

Minna Honkila[1,2], MD, PhD, Specialist in pediatrics

Natalia Miettinen MD

Tytti Pokka[2], Biostatistician

Terhi Tapiainen[1,2] Professor of Pediatrics

[1]Oulu University Hospital, Department of Children and Adolescents

[2]University of Oulu, PEDEGO research group

This document was authored by

- Suvi Mattila, MD, Doctoral student, Doctor in training (Pediatrics)

The document is hereby signed by

Author of the SAP<br><br>Signature ____________________________________<br><br>Suvi Mattila Date
Statistician<br><br>Signature ____________________________________<br><br>Tytti Pokka Date
Chief investigator<br><br>Signature ____________________________________<br><br>Professor Terhi Tapiainen Date

ABBREVIATIONS AND ANALYSIS POPULATION DEFINTION

**ED**	Emergency department
**CI**	Confidence interval
**CONSORT**	Consolidated Standards of Reporting Trials
**CSR**	Clinical study report
**PCR**	Polymerase chain reaction
**POC**	Point-of-care
**RR**	Risk ratio
**SAP**	Statistical Analysis Plan

Analysis Population Definitions

**Intervention group**	Participants allocated to receive with point-of-care diagnostic testing for respiratory pathogens on arrival at emergency department
**Control group**	Participants allocated to receive routine clinical care
**Intention-to-Treat (ITT)**	All randomized participants
**Per protocol (PP)**	Participants who received allocated intervention with no deviation to the protocol

1. INTRODUCTION

This document describes the statistical analyses and data presentations for the main paper reporting results from the single center randomized clinical trial to assess the clinical impact of multiplex PCR testing for respiratory pathogens in acutely ill children. The statistical analysis plan is based on the latest version of HeVi Trial protocol "The clinical impact of immediate identification of respiratory pathogens in acutely ill children: A randomized clinical trial" (NCT03932942).

This statistical analysis plan (SAP) provides a comprehensive and detailed description of the strategy, rationale, and statistical techniques to be used to assess the clinical impact of point-of-care multiplex PCR diagnostic for respiratory pathogens on arrival at emergency department compared with routine ED admission. The purpose of the SAP is to ensure the credibility of the study findings by specifying the statistical approaches to the analysis of study data prior to snapshot. The analysis will be carried out by an identified, appropriately qualified and experienced statistician, who will ensure the integrity of the data during the processing. The statistical analysis plan is based on the latest version of the protocol. This SAP provides additional details around the statistical analyses that are outlined in the original protocol dated Jan 23th 2019.

2 BACKGROUND INFORMATION

2.1 Rationale

During recent years, the laboratory methods based on nucleic acid amplification that can identify respiratory pathogens in respiratory secretions have developed. These multiplex panel assays can simultaneously detect up to 20 different respiratory pathogens including up to four atypical bacteria. The most recently developed platforms have a turnaround time about 1 h and due to workflow simplicity, they can be placed in pediatric emergency rooms and be used by the nurse on call. However, the evidence on expanded testing and its contribution to clinical management decisions is unclear. The aim of this trial is to evaluate the clinical utility of the point-of-care diagnostic testing of respiratory pathogens on ED admission in an unselected population of children with suspected respiratory infection.

2.2 Objectives of the trial

2.2.1. Primary objective

Primary objective is to assess if multiplex PCR testing for respiratory pathogens reduces antibiotic consumption in acutely ill children with suspected respiratory infection.

2.2.2. Secondary objectives

The secondary objectives are to investigate the effect of intervention on antibiotic prescriptions within 7 days after study entry, macrolide antibiotic prescriptions at ED visit, macrolide antibiotics started in infants under 3 months, hospital admissions during the ED visit and 7 days after study entry, diagnostic tests performed in the emergency room, outpatient telephone contacxts within 7 days after study entry, intensive care within 30 days after study entry, mortality within the next 30 days after study entry and visit associated costs.

Additional outcomes are proportion of children in whom the pathogen with targeted treatment available (influenza, *Mycoplasma pneumoniae*, *Bordetella pertussis, Legionella pneumophila*) was detected, proportion of participants receiving targeted antimicrobial treatment and time to initiation of targeted therapy.

2.3 Trial design

The trial is a single-center, open, parallelly randomized controlled trial comparing immediate point-of-care testing for respiratory tract pathogens to a control group where samples are only taken at doctor's discretion and analyzed in a central laboratory during office hours on weekdays. As we assume that the trial group will benefit from the more rapid diagnostics enabled by the adoption of

POC testing, the groups are randomized into two groups in a 2:1 ratio (point-of-care testing on arrival: routine clinical care).

2.4 Eligibility

2.4.1. Inclusion criteria

Patients are eligible for the trial in case the child's legal guardian gave written informed consent to participate **AND**

- the presence of respiratory tract symptoms (at least one of the following: cough, rhinitis, increased respiratory rate, respiratory distress, sore throat or earache) **AND/OR**
- fever of at least 38.0°C, measured either in the emergency room or at home **AND/OR**
- other suspicion of respiratory infection

Patients with comorbidities will be included.

2.4.2. Exclusion criteria

Exclusion criteria were

- the need for resuscitation at the emergency room
- the need for immediate transfer to pediatric intensive care unit

2.5. Interventions

The randomized interventions will be as follows:

2.5.1. Point-of-care test group (intervention group)

The new treatment practice studied:	***On emergency room registration, a sample is taken from children with respiratory tract symptoms and/or fever. The sample is analyzed with the POC device in the emergency room, and the result is available during the emergency room visit.***

- The sampling technique does not differ from the current practice and the sample is taken from phlegm suctioned from the patient or from the nasopharynx using a nylon or dacron swab in children with runny noses, and in unclear cases (fever with no respiratory tract symptoms), from the pharynx.

2.5.2. Control group

Current treatment practice: ***The diagnostic tests needed are conducted in the laboratory during office hours following a clinical evaluation by the doctor who prescribed the test***

At the moment (since spring 2019), the following tests for respiratory tract pathogens are available in the emergency room of Oulu University Hospital. The tests are done in the laboratory and are prescribed by the on-call physician.

- Nucleic acid detection test for **influenza and RS virus**, analyzed in the laboratory; the results are available **approximately 2–3 hours** after sampling.
- In addition, since 2019, a test covering the most common **respiratory tract viruses as well as mycoplasma and pertussis**, which is done in the laboratory **during office hours** (Qiagen, Table 2**)**
- For both tests, the sample is most commonly taken from phlegm suctioned from the patient, or from the nasopharynx using a nylon or dacron swab.

2.6 Definitions of primary and secondary outcomes

2.6.1. Primary outcome

1. Proportion of children with antibiotic prescription at emergency room
2. The pathogen-targeted antibiotic therapy is defined as antibiotic therapy (not including antivirals) prescribed to a participant in whom pathogen with specific treatment available (*M. pneumoniae, L. pneumophila or B. pertussis*) was detected.

2.6.2. Secondary outcome measures

	**Outcome**	**Definition of outcome in ClinicalTrials**
**1**	Treatment with antibiotics initiated within a week (7 days) of the emergency room visit and different groups of antibiotics	Proportion of children with antibiotics in one week
**2**	Macrolide antibiotics prescribed in the ED	Proportion of children receiving macrolide antibiotic at pediatric emergency room
**3**	Macrolide antibiotics started in infants	Proportion of infants aged < 3 months receiving

	under 3 months (proportion)	macrolide antibiotic at pediatric emergency room
**4**	Immediate admittance to hospital from emergency room (hospitalized pediatric patients)	Proportion of children admitted to hospital
**5**	Hospital admissions within a week after emergency room visit	Proportion of children admitted to hospital within one week after study entry
**6**	Other diagnostic tests performed in the emergency room	Number of other diagnostic tests than point-of-care test performed at emergency room
**7**	Revisits to emergency room within the next 7 days	Proportion of children admitted to hospital or revisit at emergency room within 7 days after study entry
**8**	Outpatient phone contacts and their number (with nurse or physician) within 7 days	Proportion of children with outpatient telephone contact within 7 days after discharge from emergency room
**9**	Ancillary laboratory testing	Number of diagnostic tests per child other than point-of-care test performed within one week
**10**	Intensive care	Proportion of children with admission to pediatric intensive care unit or intensive care unit within 7 dyas after study entry
**11**	Mortality within the next 30 days after the emergency room visit	Proportion of children who died within one month after study entry
**12**	Visit associated costs	Cost in euros per child per visits
**13**	Length of stay at ED	Length of stay at emergency room in minutes
**14**	Pathogen directed therapy	Proportion of children receiving correct pathogen directed therapy
**15**	Time from emergency room visit to onset of pathogen-directed treatment	Time to initiation of correct pathogen directed therapy

2.6.3.

Explanatory post hoc analyses

During the review process following post hoc- and subgroup analyses were requested and conducted:

1. Days of therapy (DOT), defined as the count of the number of individual antibiotic agents given to a patient on each calendar day regardless of the number of doses.
2. The proportion of participants discharged within 90 minutes after admission to emergency room will be compared.
3. Subgroup analyses to compare antibiotic prescriptions in the ED excluding those participants in the control group who underwent testing for influenza and RSV
    a. Additional analyses with adjustment for age and sec
4. Subgroup analyses to compare antibiotic prescriptions in the ED excluding those participants in the control group who underwent testing for influenza and RSV
    a. Additional analysis with adjustment for age and sex

2.7 Hypothesis framework

For each of the primary and secondary outcomes, the null hypothesis will be that there is no true difference in effect between the intervention arms.

2.8 Sample size

The annual number of visits to the pediatric emergency room at Oulu University Hospital is about 4,000–5,000; of these, about 50–70% are related to infections. The main outcome measure in the study is the number of antibiotic treatment courses initiated. We estimate that according to current treatment practice, treatment with antibiotics is started in about 25% of acutely sick children with fever in the emergency room at Oulu University Hospital. A relative reduction of 25% in the number of antibiotic treatment courses initiated was estimated as the lowest clinically significant reduction in antibiotic therapy. In the review of Doan et al. [1] (2014), a 25% reduction in antibiotic therapy and hospital treatment was considered clinically significant as well.

When the subjects were randomized to groups at a 2:1 ratio, assuming α error probability of 0.05 and power (type 1β error) of 0.8, a sample size of 1,062 subjects in the intervention group and 531 in the control group is needed, resulting in a total sample size of 1,593 subjects. In view of dropouts, we will recruit an additional 50 subjects to the new intervention group and 25 subjects to the control group, yielding a total number of 1,668 subjects recruited.

Some patients revisit the emergency room and they can be recruited again if the clinical picture has deteriorated significantly or a new illness is suspected, but only first visits are included in the targeted sample size.

It is estimated that gathering of data will take 12 months, and the trial ends when the full sample size is reached. If the targeted sample size is reached before 12 months, data gathering will continue for a total of 12 months to achieve a sample that covers a whole epidemiological year. No interim analyses will be made.

The original precise calculation was made using StatsDirect 3 software:

<u>Sample size for independent cohort study</u>

Probability of event in control group = 0.25

Probability of event in experimental group = 0.1875

Controls per case subject = 0.5

Alpha = 0.05

Power = 0.8

For uncorrected chi-square test:	N = 1,014 case subjects and 507 controls
For corrected chi-square and Fisher's exact tests:	N = **1,062** case subjects and **531** controls

Due to the current pandemic, the statistical power was calculated based on the data about antibiotic consumption in study population to decide whether to complete or discontinue the study due to Covid-19 pandemic. On Feb 18, 2020, in the study database, antibiotics had been prescribed in 30.4% of children (about 700 children). No interim analysis was performed. At the time it was estimated that the proportion of children with antibiotic prescriptions would be higher in the control group, about 33%. By using the baseline proportion 33% in the control group, and with the same relative reduction of 25%, we calculated the final sample size.

A new calculation of the sample size required was made (Feb 18, 2020) using StatsDirect 3:

<u>Sample size for independent cohort study</u>

Probability of event in control group = 0.33

Probability of event in experimental group = 0.25

Controls per case subject = 0.5

Alpha = 0.05

Power = 0.8

For uncorrected chi-square test:	N = 747 case subjects and 373 controls
For corrected chi-square and Fisher's exact tests:	**N = 785 case subjects and 392 controls (1177 children in total)**

Missing data were rare due to comprehensive medical records in the hospital and in the national registers. **Based on the calculation, the sample size reached was considered sufficient in terms of statistical power even if the randomization had to be stopped prematurely**.

2.9 Randomisation and blinding

The study is a single-center open label clinical trial, randomization is done in 2:1 permuted blocks, the size of which varies randomly between 3, 6, and 9. The randomization is done using computer-generated random sequence numbers by a biostatistician not involved in data gathering. The biostatistician draws up a list of numbers. After this, a study nurse who is not involved in the trial or data gathering places the interventions in non-transparent envelopes with a running trial number (1-1668). After randomization, the study is an open study, because the outcome measures compared are not subjective but data collected from medical records, and randomization would not be relevant in this setting. Trial design did not enable blinding.

2.10 Data collection

All randomised participants will be followed up until 30 days after randomisation.

Study physicians (SM, NP, MH) manually review all hospital medical records and Kanta services database, which is a nationwide centralized electronic database covering nearly all prescription data and medical record data in Finland [2]. Study physicians (SM, NP, MH) collect data on antibiotic prescriptions, hospitalizations, readmissions and outpatient telephone contacts and manually entered data to the statistical software. Data on laboratory tests performed and length of stay at emergency department were received from local centralized database. Visit associated costs were provided by KulasDW database, which is a database to collect expenses on healthcare visits and to charge expenses from the municipality where patient lives.

2.11 Trial reporting

The trial will be reported according to the principles of the CONSORT statements.

3 ANALYSIS POPULATIONS

3.1 Population definitions

The intention to treat (ITT) population will be all participants randomised, irrespective of intervention received.

No interim analyses are performed.

4 DESCRIPTIVE ANALYSES

4.1 Participant throughput

The flow of participants through the trial will be summarised using a CONSORT flowchart. The flowchart will describe the numbers of participants randomly allocated, who received allocation, withdrew consent, and included in the ITT analysis population.

4.2 Baseline characteristics for randomized groups

The following characteristics will be described separately for patients randomised to each arm. Differences between groups are not tested statistically.

4.3 Completeness of follow-up

Loss to follow-up is expected to be minimal as the most of data for primary and secondary outcomes is manually collected from routine clinical data.

4.4. Protocol violations or deviations

Protocol violations are classed and reported as follows [3]:

1) ***Enrolment PVs*** occurred when a member of the research team failed to appropriately apply the study's eligibility criteria resulting in the enrolment of an inappropriate patient into the trial.
    a. ***Did not fill inclusion criteria***
    b. ***Hospitalized patient recruited***
2) A ***randomisation PV*** was defined as a technical or human error leading to the violation of the intended randomisation sequence or any attempts to subvert allocation concealment.
    a. Intended randomization failed
3) **A *study intervention PV*** was defined as a dosing, timing or delivery error in the study intervention attributable to members of the research team. The research team included members of the study coordinating centre, site investigators, research coordinators and members of the healthcare team caring for participants.
    a. The sample taken from the participant in the control group was anallyzed immediately
    b. Technical error of the diagnostic device
    c. No sample taken from the participant in the intervention group

4) A *patient compliance PV* involved study participants failing to comply with the trial protocol regarding a study intervention or other requirements of participation in the trial (e.g. skipping

scheduled appointments). Formal withdrawal of consent to participate was not considered a patient compliance PV.

5) ***Data collection PVs*** encompassed errors in which the research team failed to comply with pre-specific trial guidelines for data collection and/or outcome evaluation due to avoidable reasons.

5 COMPARATIVE ANALYSES

For all outcomes, the primary analysis will be performed on the intention to treat (ITT) population. Pairwise comparisons for each outcome between randomization arms will be reported.

5.1. Primary outcome

The counts and proportions of participants to receive antibiotic prescription at ED will be reported. To analyze the primary outcomes, we will calculate 95 % confidence intervals (CI) of the differences using a Standard Normal Deviate (SND)[4] test for the proportions. Risk ratios (RR) with 95 % CI are calculated.

5.2. Secondary outcomes

5.2.1. Antibiotic prescription within 7 days after study entry

The counts and proportions of participants to receive antibiotic prescription at ED and during following 7 days will be summarized and reported. To analyze the primary outcomes, we will calculate 95 % confidence intervals (CI) of the differences using a Standard Normal Deviate (SND)[4] test for the proportions. Risk ratios (RR) with 95 % CI are calculated.

5.2.2. Macrolide antibiotic prescriptions at ED

The counts and proportions of participants to receive macrolide antibiotic prescription at ED will be reported. To analyze the primary outcomes, we will calculate 95 % confidence intervals (CI) of the differences using a Standard Normal Deviate (SND)[4] test for the proportions. Risk ratios (RR) with 95 % CI are calculated.

5.2.3. Macrolide antibiotic prescriptions in infants aged under 3 months

The counts and proportions of infants aged under 3 months to receive macrolide antibiotic prescription at ED will be reported. To analyze the outcome, we will calculate the proportion difference with 95 % confidence intervals (CI) using a Standard Normal Deviate (SND)[4] test for the proportions. Risk ratios (RR) with 95 % CI will be calculated.

5.2.4. Hospitalization at ED visit

The counts and proportions of participants admitted to hospital from ED visit in each randomization arm will be reported. To analyze the outcome, we will calculate 95 % confidence intervals (CI) of the differences using a Standard Normal Deviate (SND)[4] test for the proportions. Risk ratios (RR) with 95 % CI are calculated.

5.2.5. Hospitalization within 7 days after study entry

This outcome is defined as pediatric patients admitted to hospital within a week (7 days) of the emergency room visit. The counts and proportions of participants ad mitted to hospital during following 7 days after study entry in each randomization arm will be reported. To analyze the outcome, we will calculate 95 % confidence intervals (CI) of the differences using a Standard Normal Deviate (SND)[4] test for the proportions. Risk ratios (RR) with 95 % CI are calculated.

5.2.6. Diagnostic tests performed in the emergency room

The average sum of laboratory diagnostic test with 95 % confidence interval will be calculated for each randomization arm. Chest radiographs will be reported as proportion of participants with chest X-ray performed following 7 days after randomization. To analyze the outcome, we will calculate 95 % confidence intervals (CI) of the differences using a Standard Normal Deviate (SND)[4] test for the proportions. Risk ratios (RR) with 95 % CI are calculated.

5.2.7. Readmissions to pediatric emergency room

This outcome is defined as revisit to any emergency room within next 7 days after study entry. The counts and proportions of participants readmitted to emergency room during following 7 days after study entry in each randomization arm will be reported. To analyze the outcome, we will calculate 95 % confidence

intervals (CI) of the differences using a Standard Normal Deviate (SND)[4] test for the proportions. Risk ratios (RR) with 95 % CI are calculated.

5.2.8. Outpatient telephone contacts

This outcome will be defined as the proportion of participants with at least one emergency department visit related telephone contact to nurse or physician within following seven days after study entry. The counts and proportions of participants with telephone contact during following 7 days after study entry in each randomization arm will be reported. To analyze the outcome, we will calculate 95 % confidence intervals (CI) of the differences using a Standard Normal Deviate (SND)[4] test for the proportions. Risk ratios (RR) with 95 % CI are calculated.

5.2.9. Ancillary laboratory testing

This outcome is defined as the mean count of laboratory tests performed on each participant and proportion of participants with chest X-ray at ED or any radiological imaging within 7 days after study entry. The average sum of laboratory tests will be calculated and reported for each randomization arm. To analyze the outcome, a t-test for continuous valuables with 95 % confidence interval will be calculated. The counts and proportions of participants with chest X-ray at ED of any radiological imaging will be reported. To analyze the outcome, we will calculate 95 % confidence intervals (CI) of the differences using a Standard Normal Deviate (SND)[4] test for the proportions. Risk ratios (RR) with 95 % CI are calculated.

5.2.10. Intensive care

This outcome is defined as proportion of participants admitted to intensive care unit for any reason during following 30 days after study entry. The counts and proportions of participants admitted to intensive care unit during following 30 days after study entry in each randomization arm will be reported. To analyze the outcome, we will calculate 95 % confidence intervals (CI) of the differences using a Standard Normal Deviate (SND)[4] test for the proportions. Risk ratios (RR) with 95 % CI are calculated.

5.2.11. Mortality

This outcome is defined as proportion of participants who died for any reason during following 30 days after study entry. The counts and proportions of participants to die during following 30 days after study entry in each randomization arm will be reported. To analyze the outcome, we will calculate 95 %

confidence intervals (CI) of the differences using a Standard Normal Deviate (SND)[4] test for the proportions. Risk ratios (RR) with 95 % CI are calculated.

5.2.12. Visit associated costs

Total costs related to emergency department visit will be collected. The average sum of costs will be calculated and reported for each randomization arm. To analyze the outcome, a t-test for continuous valuables with 95 % confidence interval will be calculated.

5.2.13. Length of stay at ED

The length of stay at emergency department will be defined as time from arrival at the emergency department to discharge from emergency room or transfer to hospital ward or intensive care unit. The average length of stay at emergency department in minutes will be calculated and reported from each randomization arm. To analyze the outcome, a t-test for continuous valuables with 95 % confidence interval will be calculated.

5.2.14. Proportion of children receiving correct pathogen directed therapy

This outcome will be defined as proportion of participants with pathogen targeted treatment available in each randomization arm. The counts and proportions of participants in whom the pathogen with targeted treatment available was detected in each randomization arm will be reported. To analyze the outcome, we will calculate 95 % confidence intervals (CI) of the differences using a Standard Normal Deviate (SND)[4] test for the proportions. Risk ratios (RR) with 95 % CI are calculated.

5.2.15. Time to initiation of correct pathogen directed therapy

This outcome is defined as time to intitiation of targeted antimicrobials. This outcome is analyzed with Kaplan-Meier method using time-to-event analysis with 95 % confidence interval reported. .

5.3. Significance levels and adjustment for multiplicity

Evaluation for each outcome will be conducted independently and no adjustment will be made. Formal adjustment will not be made for multiple comparisons. 95% confidence intervals will be presented for estimates throughout the analyses.

5.4. Statistical software employed

All analyses will be performed using IBM SPSS Statistics for Windows, version 27 (Armonk, NY: IBM Corp) and StatsDirect statistical software, version 3 (England: StatsDirect Ltd).

6. SAFETY DATA

Any suspected unexpected adverse reaction will be listed by trial allocation.

7. REFERENCES

1. Doan, Q., et al., *Rapid viral diagnosis for acute febrile respiratory illness in children in the Emergency Department.* Cochrane Database Syst Rev, 2014(9): p. CD006452.

2. Jormanainen, V., *Large-scale implementation and adoption of the Finnish national Kanta services in 2010–2017: a prospective, longitudinal, indicator-based studyale implementation and adoption of the Finnish national Kanta services in 2010–2017: a prospective, longitudinal, indicator-based study.* Finnish Journal of EHealth and EWelfare, 2018. **10**(4): p. 381-395.

3. Sweetman, E.A. and G.S. Doig, *Failure to report protocol violations in clinical trials: a threat to internal validity?* Trials, 2011. **12**: p. 214.

4. Armitage P., B.G., Matthews JNS., edc., *Statistical Methods in Research*. 4th ed. 2020: Blackwell Science.

DOCUMENT HISTORY

Version	Date	Edited by	Comments
1.0	Jan 23rd 2019	SM	First draft
2.0	Mar 26th 2019	SM	Aligned with outcome updated to protocol
3.0	Apr 18th 2019	SM	Aligned with spesified outcome
4.0.	Dec 19th 2019	SM	Aligned with updated additional outcomes
5.0	Feb 12th 2020	SM	Aligned with updated protocol
6.0	Feb 18th 2020	SM	Aligned with updated protocol: sample size re-calculated
7.0	Mar 29th 2022	SM	Addition of post hoc -analyses requested in review process
