# Supplementary material for: Effect of Point-of-Care Testing for Respiratory Pathogens on Antibiotic Use in Children: A Randomized Clinical Trial
Source: JAMA Netw Open. 2022 Jun 9;5(6):e2216162. doi: 10.1001/jamanetworkopen.2022.16162 (PMC9185185; doi:10.1001/jamanetworkopen.2022.16162)
Supplement: Supplement 2. — eTable 1. Post Hoc Explanatory Analyses eTable 2. Studies on the Clinical Impact of Point-of-Care Diagnostics of Respiratory Pathogens in Acutely Ill Children With Respiratory Symptoms: Study Design, Sample Sizes, Methods and Tested Pathogens of the Previous Studies eTable 3. Studies on the Clinical Impact of Point-of-Care Diagnostics of Respiratory Pathogens in Acutely Ill Children With Respiratory Symptoms: Outcomes of the Previous Studies eReferences eFigure. Seasonality of Respiratory Pathogens During the Study [file jamanetwopen-e2216162-s002.pdf]

Supplemental Online Content

Mattila S, Paalanne N, Honkila M, Pokka T, Tapiainen T. Effect of point-of-care testing for respiratory pathogens on antibiotic use in children: a randomized clinical trial. *JAMA Netw Open*. 2022;5(6):e2216162. doi:10.1001/jamanetworkopen.2022.16162

eTable 1. Post Hoc Explanatory Analyses

eTable 2. Studies on the Clinical Impact of Point-of-Care Diagnostics of Respiratory Pathogens in Acutely Ill Children With Respiratory Symptoms: Study Design, Sample Sizes, Methods and Tested Pathogens of the Previous Studies

eTable 3. Studies on the Clinical Impact of Point-of-Care Diagnostics of Respiratory Pathogens in Acutely Ill Children With Respiratory Symptoms: Outcomes of the Previous Studies

eReferences

eFigure. Seasonality of Respiratory Pathogens During the Study

This supplemental material has been provided by the authors to give readers additional information about their work.

© 2022 Mattila S et al. *JAMA Network Open.*

eTable 1. Post Hoc Explanatory Analyses

Multiplex PCR respiratory pathogen findings available in the emergency room (intervention group) compared with no pathogen findings available (controls not tested for RSV and Influenza A and B)

	Intervention group	Control group[a]	Risk ratio (95 % CI)	aOR[b] (95% CI)
Antibiotic prescription in the ED, No. (%)	226/829 (27.3)	80/249 (32.1)	0.85 (0.69 to 1.05)	0.84 (0.62 to 1.15)
Hospitalization, No. (%)	346/829 (41.7)	96/249 (38.6)	1.08 (0.91 to 1.30)	1.10 (0.81 to 1.47)

[a] Patients in the control group tested for RSV and Influenza and results available in the emergency room were excluded.

[b] Adjusted for age and sex

Proportion of participants who were discharged from emergency room within 90 minutes after admission to emergency room.

	Intervention group	Control group	Risk ratio (95 % CI)
Early discharge from ED, No. (%)	83/829 (10)	60/414 (14.5)	0.69 (0.51 to 0.94)

Multiplex PCR results available at the time of discharge compared with controls (multiplex PCR not taken or results not available)

	Intervention group	Control group[a]	Risk ratio (95 % CI)	aOR[b] (95% CI)
Antibiotic prescription in the ED, No. (%)	211/764 (27.6)	118/414 (28.5)	0.97 (0.80 to 1.17)	0.98 (0.75 to 1.29)
Hospitalization, No. (%)	287/764 (37.6)	177/414 (42.8)	0.88 (0.76 to 1.02)	0.84 (0.66 to 1.08)

[a] Patients in the intervention group in whom multiplex PCR results were not available at the time of discharge were excluded.

[b] Adjusted for age and sex

© 2022 Mattila S et al. *JAMA Network Open.*

eTable 2. Studies on the Clinical Impact of Point-of-Care Diagnostics of Respiratory Pathogens in Acutely Ill Children With Respiratory Symptoms: Study Design, Sample Sizes, Methods and Tested Pathogens of the Previous Studies

**Study**	**Study design**	**Sample size**	**Age**	**Method**	**Pathogens tested**	**Results available (min)**	**Results available prior to ED visit**
**Bonner et al.[1] 2003**	Single-center RCT	391	2 months to 21 years<br><br>Data analyzed separately in age group 2–36 months with similar results	**Antigen test**<br><br>FluOIA (optical immunoassay)	Influenza	20–25	+
**Esposito et al.[2] 2003**	Single-center RCT	957	0–15 years	**Antigen test** (QuickVue Influenza test)	Influenza	10	+
**Abanses et al.[3] 2006**	Prospective case-control<br><br>Randomization failed, and the data were analyzed as a convenience sample	1007	3 months to 3 years	**Antigen test**<br><br>Directigen Flu A+B	Influenza	**--**	+
**Iyer et al.[4] 2006**	Quasi-randomized controlled study<br><br>(the method of testing was alternated by day)	700	2 months to 2 years	**Antigen test** (QuickVue Influenza test)	Influenza	Up to 30 minutes	+
**Poehling et al.[5] 2006**	Cluster randomized RCT<br>(Study days were randomized to rapid test or no rapid test days)	468	Up to 5 years	**Antigen test** (QuickVue Influenza test)<br><br>(All participants in both groups were tested with viral culture and PCR as the criterion standard)	Influenza	Not reported	+

**Doan et al.**[6] **2009**	Single-center RCT	200	3 to 36 months	Rapid respiratory virus panel, **direct immunofluorescence assay** (SimulFluor respiratory screening agent)	Adenovirus, Influenza A and B, Parainfluenza 1, 2 and 3, RSV	30–150 (rapid test available only during laboratory hours (8 am to 9 pm during weekdays and 8 am to 6:30 pm during weekends)	+
**Wishaupt et al.**[7] **2011**	A controlled clinical trial;<br><br>Samples were randomized in the laboratory: samples with even order numbers were assigned to the intervention group and samples with odd numbers were assigned to the control group	583	Up to 12 years	RT-PCR	Intervention:<br>RSV A, RSV B, influenza viruses A and B, adenovirus, parainfluenza viruses 1, 2, 3, and 4, human bocavirus, coronaviruses 229E, OC43, and NL63, human metapneumovirus, rhinovirus, Chlamydophila pneumoniae, Mycoplasma pneumonia, *and* Bordetella pertussis<br><br>All patients: **DFA**s for RSV, influenza A and B, adenovirus, and parainfluenza viruses 1, 2, and 3 and viral culture	Following day	Results reported on the following day
**Echavarria et al.**[8] **2018**	A randomized non-blinded study with 156 children and 276 adults	156	2 months to 6 years	FilmArray Respiratory Panel compared to indirect immunofluorescence assay	Intervention (FilmArray-RP): RSV, FluA H1, H1-2009, H3, FluB, AdV, PIV 1–4, RV/EV, HMPV, HCoV OC43, 229E, NL63, HKU1, Bordetella pertussis, Mycoplasma pneumoniae, and Chlamydia pneumoniae<br>*Control (IFA):* RSV, FluA, FluB, PIV 1–3, and AdV	Intervention: 65 minutes<br>Control: samples batched once a day and results reported by 4:00 each day	
**Schechter-Perkins et al.**[9] **2019**	Single center randomized controlled trial	197	4 months to 82 years	multiplex real time Rt-PCR assay	Influenza A/B	20 minutes (Subjects enrolled Monday	+

© 2022 Mattila S et al. *JAMA Network Open.*

				(Cobas Liat Influenza A/B device)		to Friday, 8 am to 11 pm)	
**Kitano et al.[10] 2020**	Retrospective observational study of hospitalized children with a retrospective cohort as a control group	1281 (1132 with rapid antigen test and 149 with multiplex-PCR)	Not reported	The FilmArray multiplex PCR respiratory panel compared to rapid antigen test	Multiplex PCR: Adenovirus, Coronavirus HKU1, NL63, 229E or OC43, Human metapneumovirus, Rhinovirus or Enterovirus, Influenza virus A, A H1, A H3, A H1-2009 or B, Respiratory syncytial virus, Parainfluenza virus 1, 2, 3 or 4, Mycoplasma pneumoniae, Chlamydia pneumoniae, Bordetella pertussis.<br><br>RAT: rapid antigen test for influenza, human metapneumovirus, respiratory syncytial virus, adenovirus, Group A Streptococcus, and Mycoplasma pneumoniae available	60 minutes	Multiplex PCR available weekdays from 8:30 to 17:15
**Reischl et al.[11] 2020**	Retrospective observational study of children admitted to the infectious disease ward with retrospective cohort as a control group	786	Up to 16 years	RT-PCR<br><br>(FA Respiratory panel)	Adenovirus, coronavirus 229E, coronavirus HKU1, coronavirus NL63, coronavirus OC43, human metapneumovirus, influenza A, influenza A subtype H1, influenza A subtype H3, influenza A subtype H1-2009, influenza B, parainfluenza virus 1, parainfluenza virus 2, parainfluenza virus 3, parainfluenza virus 4, human rhinovirus/enterovirus, respiratory syncytial virus, Bordetella pertussis, Chlamydophila pneumoniae, and Mycoplasma pneumoniae	Two working days	-
**Rao et al. [12] 2021**	A randomized controlled trial	920	1 mo to 18 years	BioFire FilmArray RP2 Panel	Adenovirus, coronavirus 229E, coronavirus HKU1, coronavirus NL63, coronavirus OC43, human metapneumovirus, influenza A, influenza A subtype H1, influenza A subtype H3, influenza A subtype H1-2009, influenza B,	45 min	modified ITT analyses whereby the intervention group comprised children whose clinicians knew the RRP test

© 2022 Mattila S et al. *JAMA Network Open.*

					parainfluenza virus 1, parainfluenza virus 2, parainfluenza virus 3, parainfluenza virus 4, human rhinovirus/enterovirus, respiratory syncytial virus, Bordetella pertussis, Chlamydophila pneumoniae, and Mycoplasma pneumoniae		results vs test results not available for the clinician

© 2022 Mattila S et al. *JAMA Network Open.*

eTable 3. Studies on the Clinical Impact of Point-of-Care Diagnostics of Respiratory Pathogens in Acutely Ill Children With Respiratory Symptoms: Outcomes of the Previous Studies

Study	Antibiotic prescription rate	Antiviral prescription rate	Hospital admission rate	Length of ED visit	Readmission to ED	Rate of ancillary tests	Cost efficiency
**Bonner et al.[1] 2003**<br><br>Data analyzed separately in age group 2–36 months with similar results	*Influenza positive MD aware (n = 96) vs. MD unaware (n = 106)*<br><br>**7/26 vs. 26/106 p = < 0.001**	*Influenza positive MD aware (n = 96) vs. MD unaware (n = 106)*<br><br>**18 vs. 7, p = 0.02**	Not reported	*Influenza positive MD aware (n = 96) vs. MD unaware (n = 106)*<br><br>**25 vs. 49 min (mean time from examination to discharge) p < 0.001**	Not reported	*Influenza positive MD aware (n = 96) vs. MD unaware (n = 106)*<br><br>**CBC 0 vs. 13, p < 0.001**<br>**Blood culture 0 *vs.* 11, p < 0.001**<br>**Urinalysis 2 *vs.* 12, p = 0.011**<br>**Urine culture 3 *vs.* 14, p = 0.011**<br>Cerebrospinal fluid study/culture<br>0 *vs.* 2, p = 0.499<br>**Chest radiograph 7 *vs.* 26, p = 0.001**	*Influenza positive MD aware (n = 96) vs. MD unaware (n = 106)*<br><br>(mean charge/patient, laboratory and radiograph)<br>**$15.65 vs. 92.37, p < 0.001**
	Influenza negative MD aware (n = 97) vs. MD unaware (n = 92)<br><br>27/97 *vs.* 27/92<br>p = 0.818	*Influenza negative MD aware (n = 97) vs. MD unaware (n = 92)*<br><br>0 *vs.* 2, p = 0.236		*Influenza negative MD aware (n = 97) vs. MD unaware (n = 92)*<br><br>45 *vs.* 42 min, p = 0.549		*Influenza negative MD aware (n = 97) vs. MD unaware (n = 92)*<br><br>CBC 13 *vs.* 7, p = 0.196<br>Blood culture 12 *vs.* 6, p = 0.172<br>Urinalysis 10 *vs.* 8, p = 0.706<br>Urine culture 12 *vs.* 5 p = 0.096<br>Cerebrospinal fluid study/culture<br>3 *vs.* 2, p = 0.695<br>Chest radiograph 22 *vs.* 23, p = 0.708	*Influenza negative MD aware (n = 97) vs. MD unaware (n = 92)*<br><br>$93.07 *vs.* $68.91 p = 0.871

**Esposito et al.[2] 2003**<br><br>At the time of the study, no antiviral drug was approved for use in the therapy of influenza in children	*Influenza positive (n = 43) vs. influenza negative (n = 435)*<br><br>**32.6% vs. 64.8% p < 0.0001**<br><br>*Influenza positive (n = 43) vs. no test (n = 479)*<br><br>**32.6% *vs.* 61.8%, p = 0.0003**	*Influenza positive (n = 43) vs. influenza negative (n = 435)*<br><br>0 *vs.* 0%<br><br>*Influenza positive (n = 43) vs. no test (n = 479)*<br><br>0 *vs.* 0%	*Influenza positive (n = 43) vs influenza negative (n = 435)*<br><br>0 *vs.* 4.6% p = 0.240<br><br>*Influenza positive (n = 43) vs no test (n = 479)*<br><br>0 *vs.* 5.8% p = 0.154	Not reported	Not reported	*Influenza positive (n = 43) vs. influenza negative (n = 435)*<br><br>**Blood examination**<br>**2.3% *vs.* 14.5%, p = 0.045**<br>Chest radiograph<br>4.6% *vs.* 11.7%, p = 0.207<br><br>*Influenza positive (n = 43) vs. no test (n = 479)*<br>**Blood examination**<br>**2.3% *vs.* 15.0% p = 0.038**<br>Chest radiograph<br>4.6% *vs.* 11.7% p = 0.208	Not reported
**Abanses et al.[3] 2006**<br><br>Randomization failed and the data was analyzed as a convenience sample	Standard protocol (n = 719) *vs.* tested in triage (n = 288)<br><br>30% *vs.* 35%,<br>RR 0.84 (0.70–1.02)	Not reported	Not reported	Standard protocol (n = 719) *vs.* tested in triage (n = 288)<br><br>185+/- 80 min *vs.* 185 +/-86, (CI for mean difference<br>-12 -12)	Not reported	Standard protocol (n = 719) *vs.* tested in triage (n = 288)<br><br>CBC 22 *vs.* 17% RR 1.3 (95% CI 0.95–1.7)<br><br>BC 21% *vs.* 17% RR 1.2 (95% CI 0.95–1.7)<br><br>RSV testing 18% *vs.* 7.3%, RR 2.5 (1.6–3.9)<br><br>UA 18% *vs.* 14%, RR 1.4 (0.98–1.9)<br><br>LP 0.3% *vs.* 0%<br><br>**CXR 26% *vs.* 20%, RR 1.3 (1.01–1.7)**	Standard protocol (n = 719) *vs.* tested in triage (n = 288)<br><br>Total charges ($)<br>544 +/- 358 *vs.* 538 +/- 427, CI for mean difference

© 2022 Mattila S et al. *JAMA Network Open.*

**Iyer et al.[4] 2006**	Point of care (n = 345) *vs.* standard test (n = 355) (95% CI)<br><br>Antibiotics given in the ER<br>15.7% (11.8, 19.5) *vs.* 16.6% (12.7, 20.5)<br><br>Discharged with antibiotic prescription<br>25.3% (20.3, 30.2) *vs.* 30.5% (25.4, 35.6)	Not reported	11.6% (8.2, 15.0) *vs.* 10.4% (7.2, 13.6)	203.6 min (194.4, 212.9) *vs.* 204.1 min (194.5, 213.8)	Revisit to ED within 14 days<br><br>17.7% (13.6, 21.7) *vs.* 15.8% (12.0, 19.6)	Blood culture 24.1% (19.5, 28.6) *vs.* 27.9% (23.2, 32.6)<br>CBC 25.5% (20.9, 30.1) *vs.* 29.3% (24.5, 34.1)<br>Urinalysis 20.9% (16.6, 25.2) *vs.* 21.1% (16.9, 25.4)<br>Urine culture 20.3% (16.0, 24.6) *vs.* 20.9% (16.6, 25.1)<br>Lumbar puncture 2.0% (0.5, 3.5) *vs.* 1.4% (0.2, 2.6)<br>Chest radiograph 24.9% (20.3, 29.5) *vs.* 28.2% (23.5, 32.9)	625 $ (566, 692) *vs.* 592 $ (540, 650)
**Poehling et al.[5] 2006**	Emergency department<br>Rapid test (n = 135) *vs.* no rapid test (n = 170)<br>39% *vs.* 52%, p = 0.03<br><br>32% *vs.* 29%, p = 0.57<br><br>Acute care clinic<br>Rapid test (n = 70 *vs.* No rapid test (n = 93)<br><br>26% *vs.* 29%, p = 0.75	Emergency department<br>Rapid test (n = 135) *vs.* no rapid test (n = 170)<br>39% *vs.* 52%, p = 0.03<br><br>1% *vs.* 0 p = 0.44<br><br>Acute care clinic<br>Rapid test (n = 70 *vs.* No rapid test (n = 93)<br><br>0 *vs.* 0%	Not reported	Not reported	Not reported	Emergency department<br>Rapid test (n = 135) *vs.* no rapid test (n = 170)<br>Any diagnostic test<br>39% *vs.* 52%, p = 0.03<br>Chest radiograph 23% *vs.* 33%, p = 0.06<br>Blood count/culture 10% *vs.* 18% p = 0.05<br>Urinalysis 13% *vs.* 16%, p = 0.53<br><br>Acute care clinic Rapid test (n = 70 *vs.* No rapid test (n = 93)<br>Any diagnostic test<br>17% *vs.* 13%, p = 0.45<br>Chest radiograph 9% *vs.* 5%, p = 0.42<br>Blood count/culture 4% *vs.* 3% p = 1.00<br>Urinalysis 7% *vs.* 3%, p = 0.29	Not reported

© 2022 Mattila S et al. *JAMA Network Open.*

**Doan et al.[6] 2009**	VIRAP (n = 89) *vs.* control (n = 110)<br><br>18% *vs.* 20.9% RR 0.86, 95% CI (0.48, 1.53)<br>**Post ED antibiotic within 7 to 10 days 5.6% vs. 15.5% RR 0.36 (0.14, 0.95)**	Not reported	Not reported	105.7 (188.04) min *vs.* 156.1 (235.82) min, mean difference -50.4 (-104.6, 3.7)	Within 7 to 10 days<br>33.7% *vs.* 39.1%, RR 0.86 (0.59–1.25)	Chest X-ray 23.6% *vs.* 33.6%, RR 0.70 (0.44, 1.11)<br>Blood work<br>10.1% *vs.* 17.3%, RR 0.59 (0.28, 1.23)<br>Urinalysis 31.5% *vs.* 28.2%, RR 1.12 (0.73–1.71)<br><br>Post ED ancillary test<br>1.1% *vs.* 5.5%, RR 0.21 (0.03, 1.7)	Not reported
**Wishaupt et al.[7] 2011**	Intervention (n = 298) *vs.* control (n = 285)<br><br>**41.6% vs. 27.4% p = 0.000**	74.8% *vs.* 74% p = 0.825	Not reported	time in hospital (days)<br>3.68 +/-2.68 *vs.* 3.96+/-2.67 p = 0.170	Not reported	Not reported	Not reported
**Echavarria et al.[8] 2018**	**Reported as change in original treatment plan: 54.8% vs. 13.9% p = 0.001**<br><br>**Decrease in antibiotic prescriptions 23% vs. 2.3% p = 0.001**	Decrease and increase in oseltamivir 1.8% *vs.* 2.3%, p = 0.621	17.7% *vs.* 20.9%, p = 0.643	Length of stay at hospital<br>3 *vs.* 5 days, p = 0.218	Not reported	**Decrease in complementary studies 25.7% vs. 4.7%**<br>**OR 6.84 (95% CI 1.53–30.49)**	Not reported
**Schechter-Perkins et al.[9] 2019**	Core lab (n = 97) *vs.* point of care (n = 100)<br><br>14.4% *vs.* 14.0%, p = 0.93	Not reported	Not reported	185.9 (110.0) *vs.* 168.9 (91.7), p = 0.26	Not reported	Not reported	Not reported
**Kitano et al.[10] 2020**	**Days of therapy 8.56 vs. 12.82 p < 0.001**	-	-	Length of stay at hospital (days)<br>6.83 *vs.* 8.18, p = 0.032	Not reported	Not reported	Hospitalization costs 1286.6$ *vs.* 1421,4 $<br>Cost of tests 183,1 $ vs 39.3 $-

© 2022 Mattila S et al. *JAMA Network Open.*

**Reischl et al.[11] 2020**	Study group (n = 322) *vs.* control group (n = 464)<br><br>45% *vs.* 42.5% p = 0.784	1.9% *vs.* 0.9%	Not reported	Length of stay at hospital (days) mean 4.7 +/- 5.4 *vs.* 4.7 +/- 4.4	Not reported	Not reported	Not reported
**Rao et al.[12] 2021**	115 (25%) vs 88 (19%), RR (95% CI) 1.3 (1.0-1.7), aRR (95% CI) 1.1 (0.9-1.3)	31 (7%) vs 25 (5%), RR (95% CI) 1.1 (0.8-2.1), aRR (95% CI) 2.5 (1.5-4.2)	77 (17%) vs 69 (15%), RR (95% CI) 1.1 (0.8-1.5), aRR (95% CI) 2.0 (1.5-2.7)	3.1 vs 2.8 h	within 10 days 29 (6%) VS 29 (6%) RR (95% CI) 1.0 (0.6-1.7), aRR (95% CI) 0.9 (0.6-1.6)	Not reported	Not reported

© 2022 Mattila S et al. *JAMA Network Open.*

eReferences

1. Bonner AB, Monroe KW, Talley LI, Klasner AE, Kimberlin DW. Impact of the rapid diagnosis of influenza on physician decision-making and patient management in the pediatric emergency department: results of a randomized, prospective, controlled trial. Pediatrics 2003;112(2):363-7. DOI: 10.1542/peds.112.2.363.

2. Esposito S, Marchisio P, Morelli P, Crovari P, Principi N. Effect of a rapid influenza diagnosis. Arch Dis Child 2003;88(6):525-6. DOI: 10.1136/adc.88.6.525.

3. Abanses JC, Dowd MD, Simon SD, Sharma V. Impact of rapid influenza testing at triage on management of febrile infants and young children. Pediatr Emerg Care 2006;22(3):145-9. DOI: 10.1097/01.pec.0000202454.19237.b0.

4. Iyer SB, Gerber MA, Pomerantz WJ, Mortensen JE, Ruddy RM. Effect of point-of-care influenza testing on management of febrile children. Acad Emerg Med 2006;13(12):1259-68. DOI: 10.1197/j.aem.2006.07.026.

5. Poehling KA, Zhu Y, Tang YW, Edwards K. Accuracy and impact of a point-of-care rapid influenza test in young children with respiratory illnesses. Arch Pediatr Adolesc Med 2006;160(7):713-8. DOI: 10.1001/archpedi.160.7.713.

6. Doan QH, Kissoon N, Dobson S, et al. A randomized, controlled trial of the impact of early and rapid diagnosis of viral infections in children brought to an emergency department with febrile respiratory tract illnesses. J Pediatr 2009;154(1):91-5. DOI: 10.1016/j.jpeds.2008.07.043.

7. Wishaupt JO, Russcher A, Smeets LC, Versteegh FG, Hartwig NG. Clinical impact of RT-PCR for pediatric acute respiratory infections: a controlled clinical trial. Pediatrics 2011;128(5):e1113-20. DOI: 10.1542/peds.2010-2779.

8. Echavarria M, Marcone DN, Querci M, et al. Clinical impact of rapid molecular detection of respiratory pathogens in patients with acute respiratory infection. J Clin Virol 2018;108:90-95. DOI: 10.1016/j.jcv.2018.09.009.

9. Schechter-Perkins EM, Mitchell PM, Nelson KP, et al. Point-of-care influenza testing does not significantly shorten time to disposition among patients with an influenza-like illness. Am J Emerg Med 2019;37(5):873-878. DOI: 10.1016/j.ajem.2018.08.005.

10. Kitano T, Nishikawa H, Suzuki R, et al. The impact analysis of a multiplex PCR respiratory panel for hospitalized pediatric respiratory infections in Japan. J Infect Chemother 2020;26(1):82-85. DOI: 10.1016/j.jiac.2019.07.014.

11. Reischl AT, Schreiner D, Poplawska K, et al. The clinical impact of PCR-based point-of-care diagnostic in respiratory tract infections in children. J Clin Lab Anal 2020;34(5):e23203. DOI: 10.1002/jcla.23203.

12. Rao S, Lamb MM, Moss A, et al. Effect of rapid respiratory virus testing on antibiotic prescribing among children presenting to the emergency department with acute respiratory illness: A randomized controlled trial. JAMA Netw Open

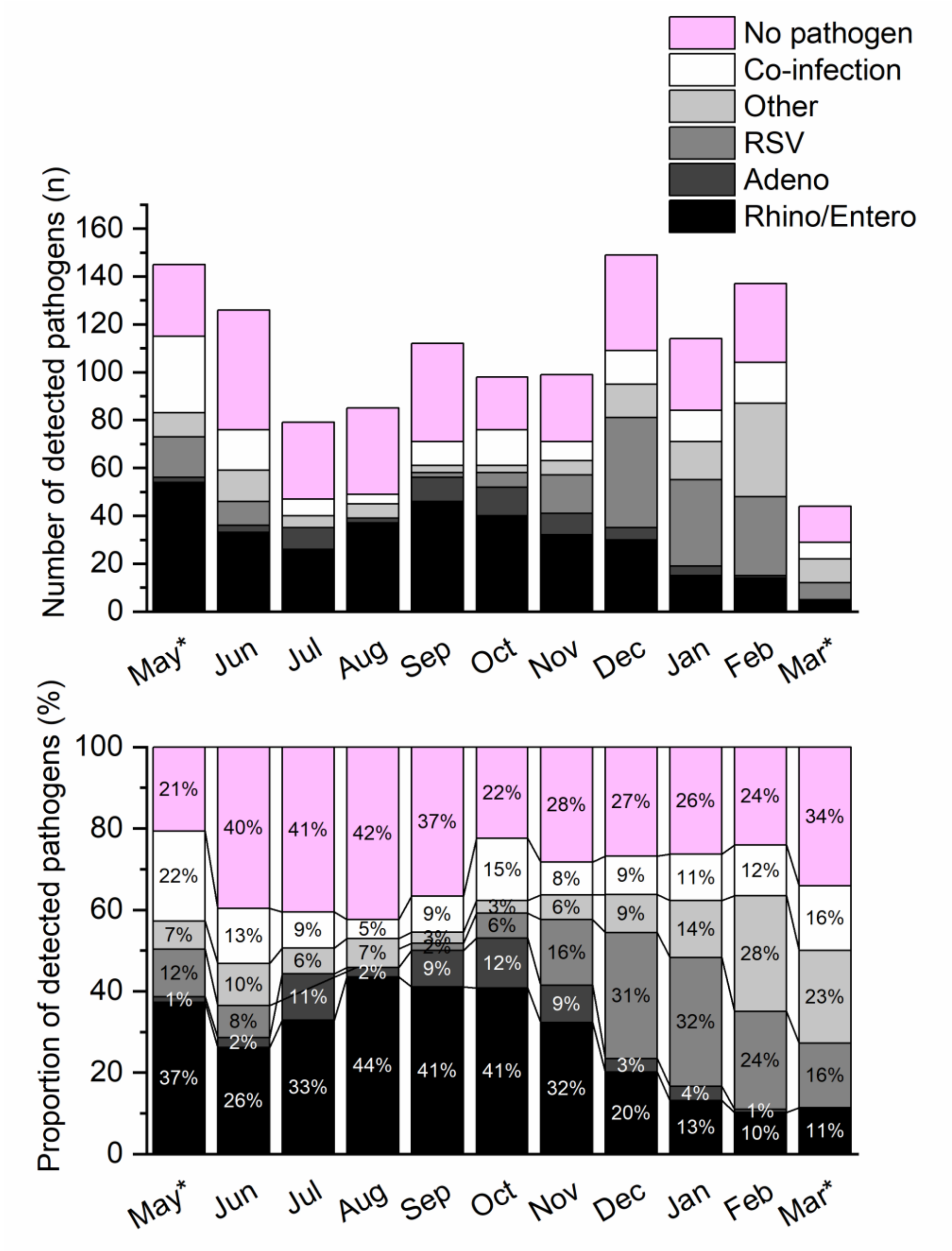

eFigure. Seasonality of Respiratory Pathogens During the Study

Both the absolute numbers and the proportions of detected pathogens are shown.
