# Supplementary material for: Effect of Point-of-Care Testing for Respiratory Pathogens on Antibiotic Use in Children: A Randomized Clinical Trial
Source: JAMA Netw Open. 2022 Jun 9;5(6):e2216162. doi: 10.1001/jamanetworkopen.2022.16162 (PMC9185185; doi:10.1001/jamanetworkopen.2022.16162)
Supplement: Supplement 3. — Data Sharing Statement [file jamanetwopen-e2216162-s00.pdf]

Data Sharing Statement

Mattila. Effect of Point-of-Care Testing for Respiratory Pathogens on Antibiotic Use in Children. *JAMA Netw Open*. Published June 09, 2022. doi:10.1001/jamanetworkopen.2022.16162

Data
Data available: Yes
Data types: Deidentified participant data
How to access data: Data sharing is possible from the principal investigator Terhi Tapiainen, Oulu University Hospital, Finland, terhi.tapiainen@oulu.fi
When available: With publication

Supporting Documents
Document types: None

Additional Information
Who can access the data: For clinical researchers for study purposes after an reasonable request.
Types of analyses: For predefined specified purposes such as individual meta-analysis of the topic.
Mechanisms of data availability: After approval of a proposal and after a signed data access agreement.
Any additional restrictions: All data elements that might reveal the identity of the participants directly or indirectly will be removed.
